# Increased joint loading induces subchondral bone loss of the temporomandibular joint via the RANTES-CCRs-Akt2 axis

**DOI:** 10.1172/jci.insight.158874

**Published:** 2022-11-08

**Authors:** Shi-Yang Feng, Jie Lei, Yu-Xiang Li, Wen-Ge Shi, Ran-Ran Wang, Adrian Ujin Yap, Yi-Xiang Wang, Kai-Yuan Fu

**Affiliations:** 1 Center for Temporomandibular Disorders & Orofacial Pain, and; 2 Central Laboratory, Peking University School and Hospital of Stomatology, Beijing, China.; 3 National Center of Stomatology & National Clinical Research Center for Oral Diseases & National Engineering Research Center of Oral Biomaterials and Digital Medical Devices, Beijing, China.; 4 State Key Laboratory of High Performance Ceramics and Superfine Microstructure, Shanghai Institute of Ceramics, Chinese Academy of Sciences, Shanghai, China.; 5 Center of Materials Science and Optoelectronics Engineering, University of Chinese Academy of Sciences, Beijing, China.; 6 School of Chemistry and Materials Science, Hangzhou Institute for Advanced Study, University of Chinese Academy of Sciences, Hangzhou, China.; 7 Department of Dentistry, Ng Teng Fong General Hospital and Faculty of Dentistry, National University Health System, Singapore, Singapore.; 8 National Dental Research Institute Singapore, National Dental Centre Singapore and Duke-NUS Medical School, Singapore Health Services, Singapore, Singapore.

**Keywords:** Bone Biology, Chemokines, Osteoarthritis, Osteoclast/osteoblast biology

## Abstract

Early-stage temporomandibular joint osteoarthritis (TMJOA) is characterized by excessive subchondral bone loss. Emerging evidence suggests that TMJ disc displacement is involved, but the pathogenic mechanism remains unclear. Here, we established a rat model of TMJOA that simulated disc displacement with a capacitance-based force-sensing system to directly measure articular surface pressure in vivo. Micro-CT, histological staining, immunofluorescence staining, IHC staining, and Western blot were used to assess pathological changes and underlying mechanisms of TMJOA in the rat model in vivo as well as in RAW264.7 cells in vitro. We found that disc displacement led to significantly higher pressure on the articular surface, which caused rapid subchondral bone loss via activation of the RANTES–chemokine receptors–Akt2 (RANTES-CCRs-Akt2) axis. Inhibition of RANTES or Akt2 attenuated subchondral bone loss and resulted in improved subchondral bone microstructure. Cytological studies substantiated that RANTES regulated osteoclast formation by binding to its receptor CCRs and activating the Akt2 pathway. The clinical evidence further supported that RANTES was a potential biomarker for predicting subchondral bone loss in early-stage TMJOA. Taken together, this study demonstrates important functions of the RANTES-CCRs-Akt2 axis in the regulation of subchondral bone remodeling and provides further knowledge of how disc displacement causes TMJOA.

## Graphical abstract

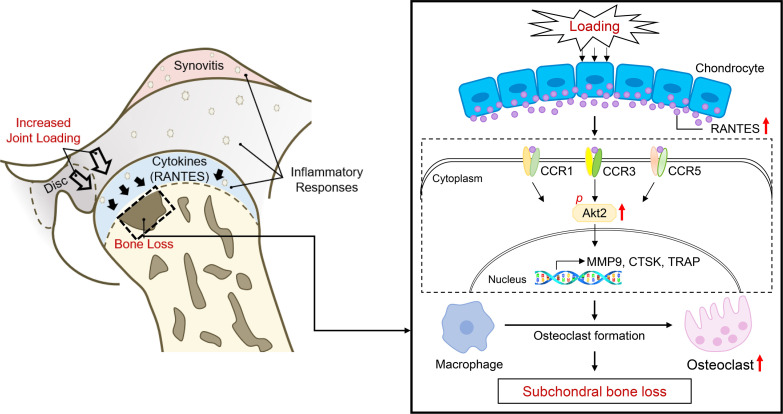

## Introduction

Osteoarthritis (OA) is the most common degenerative joint disease and the leading cause of physical disability (1). In the early stage, joint deterioration is characterized by synovial inflammation, progressive cartilage degradation, and increased bone remodeling (2–6). Although the pathogenesis of synovium and cartilage changes has been extensively investigated, little is known about the role of subchondral bone remodeling in OA. As for the temporomandibular joint (TMJ), which undergoes active bone remodeling throughout its lifetime, subchondral bone remodeling in early-stage temporomandibular joint osteoarthritis (TMJOA) is dominated by condylar surface erosion, trabecular bone loss, and reduced bone mineral density (BMD) (6–8). Recent studies have demonstrated that the early loss of subchondral bone may further cause alterations in joint shape and load transmission that predispose patients to progressive cartilage degeneration (9–11). In addition, OA therapeutics targeting subchondral bone and preventing bone mass loss have exhibited significant efficacies for treating OA, suggesting an important role of subchondral bone in the development of OA (12–16). However, the mechanisms underlying bone turnover remain unclear, especially in early-stage OA.

The initiation of subchondral bone loss involves a series of events related to mechanical joint overloading (5, 17–19). For TMJ, subchondral bone loss rapidly after internal derangement such as disc displacement without reduction (DDw/oR) (20). Using high-resolution cone-beam CT (CBCT), recent-onset DDw/oR was found to be related to subchondral bone loss in early-stage TMJOA (21). The displaced disc could interfere with condylar mobility and lead to increased joint loading on the articular surface (20). Thus, the in situ and real-time detection of mechanical joint loading is central to understanding the pathogenesis of TMJOA and its early intervention. However, to date, few studies have directly assessed and quantified loads on the articular surface and clarified the underlying mechanisms in early-stage TMJOA, owing to the lack of appropriate animal models and joint load–measurement systems.

Abnormal mechanical joint loading could trigger a cascade of bone changes involving dysregulated cellular metabolism of osteoclasts, osteoblasts, and osteocytes. Increased levels of many proinflammatory cytokines, such as interleukin receptor activator of nuclear factor κ-B ligand (RANKL), IL-6, and prostaglandin E2, promote osteoclast formation and further cause excessive bone resorption (10, 22). In our previous study, we screened for the presence of various inflammatory-related cytokines and determined that levels of RANTES were increased in the synovial fluid of patients with TMJOA (23). Other studies have focused primarily on the role of RANTES in synovial inflammatory response and extracellular matrix degradation of cartilage (24, 25), while our earlier work verified that RANTES could induce macrophage migration, osteoclast differentiation, and bone resorption in vitro (23). However, the downstream molecules of RANTES in regulating osteoclast differentiation are unknown.

In this study, we aimed to determine if increased joint loading produces subchondral bone loss and to elucidate the underlying mechanisms, using a rat model of TMJOA that was induced by disc displacement. Findings from the rat model were subsequently verified in vitro with pressurized tissue cultures as well as cytology studies and further supported by clinical evidence obtained from patients with TMJOA after disc displacement. The research hypotheses were as follows: (a) TMJ disc displacement increases joint loading and causes subchondral bone loss; (b) subchondral bone loss in early-stage TMJOA is mediated by the chemokine RANTES, which promotes osteoclast formation; (c) RANTES inhibition reduces subchondral bone loss; and (d) RANTES can serve as a possible biomarker for early-stage TMJOA.

## Results

### Disc displacement causes subchondral bone loss in early-stage TMJOA.

**Figure 1:**
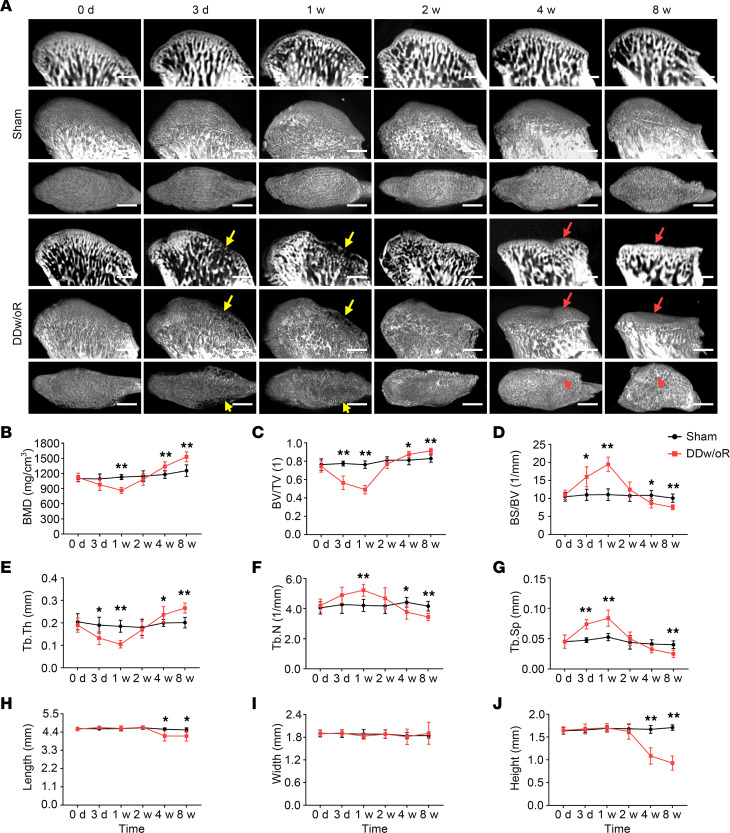
Disc displacement causes subchondral bone loss in early-stage TMJOA. Male Sprague-Dawley rats underwent sham or right-sided unilateral DDw/oR surgery. Rats were euthanized at baseline (0 days), 3 days, and 1, 2, 4, and 8 weeks after surgery. (**A**) Micro-CT images of TMJ condyles. Lines 1 and 4: sagittal view in 2-dimensional images; lines 2 and 5: sagittal view in 3-dimensional images; lines 3 and 6: horizontal view in 3-dimensional images. The yellow arrows show condylar surface erosion. The red arrows indicate condylar surface flattening and local sclerosis. Scale bar: 1 mm. (**B–G**) Quantitative analysis of (**B**) BMD, (**C**) BV/TV, (**D**) BS/BV, (**E**) Tb.Th, (**F**) trabecular number (Tb.N), and (**G**) Tb.Sp in subchondral bone of TMJ condylar heads determined by micro-CT measurements. (**H–J**) Quantitative analysis of (**H**) length, (**I**) width, and (**J**) height of TMJ condylar heads determined by micro-CT measurements. Data are presented as mean ± 95% CI, and 1 representative image of 8 independent samples per group is shown. Statistical analyses were determined by 2-way ANOVA with Bonferroni’s multiple comparison test. *P < 0.05, **P < 0.01.

To determine whether TMJ DDw/oR causes subchondral bone loss and TMJOA, we established a rat model with DDw/oR and investigated the process of condylar bone remodeling during TMJOA pathology. In the animal model, after surgical disc displacement, rats exhibited condylar subchondral bone loss rapidly that resembled early-stage TMJOA in humans (Figure 1A). Discontinuity of the condylar cortex was observed in the DDw/oR group as early as 3 days after surgery, compared with the sham-operation group. Subchondral bone loss persisted up to 1 week after surgery in the DDw/oR group and manifested as condylar surface erosion and local bone lesions, which are considered early-stage TMJOA bone changes (Figure 1A). Micro-CT analysis showed significantly lower values of BMD, the ratio of bone volume to tissue volume (BV/TV), and trabecular thickness (Tb.Th), but higher values for the ratio of bone surface area to (BS/BV), trabecular number, and trabecular separation (Tb.Sp) (Figure 1, B–G). Subsequently, subchondral bone formation, presenting as condylar surface repair and local sclerosis, was observed 2 weeks after surgery in the DDw/oR group and sustained for at least 8 weeks after surgery, shown as condylar surface flattening, extensive sclerosis, and joint deformity, which are considered late-stage TMJOA bone changes (Figure 1A). Compared with the sham-operation group, there were significantly higher values of BMD, BV/TV, and Tb.Th at the end of 8 weeks after surgery in the DDw/oR group (Figure 1, B–G). Concomitantly, the length and height of condylar heads were significantly decreased, indicating condylar deformity at 8 weeks after surgery in the DDw/oR group (Figure 1, H–J).

**Figure 2:**
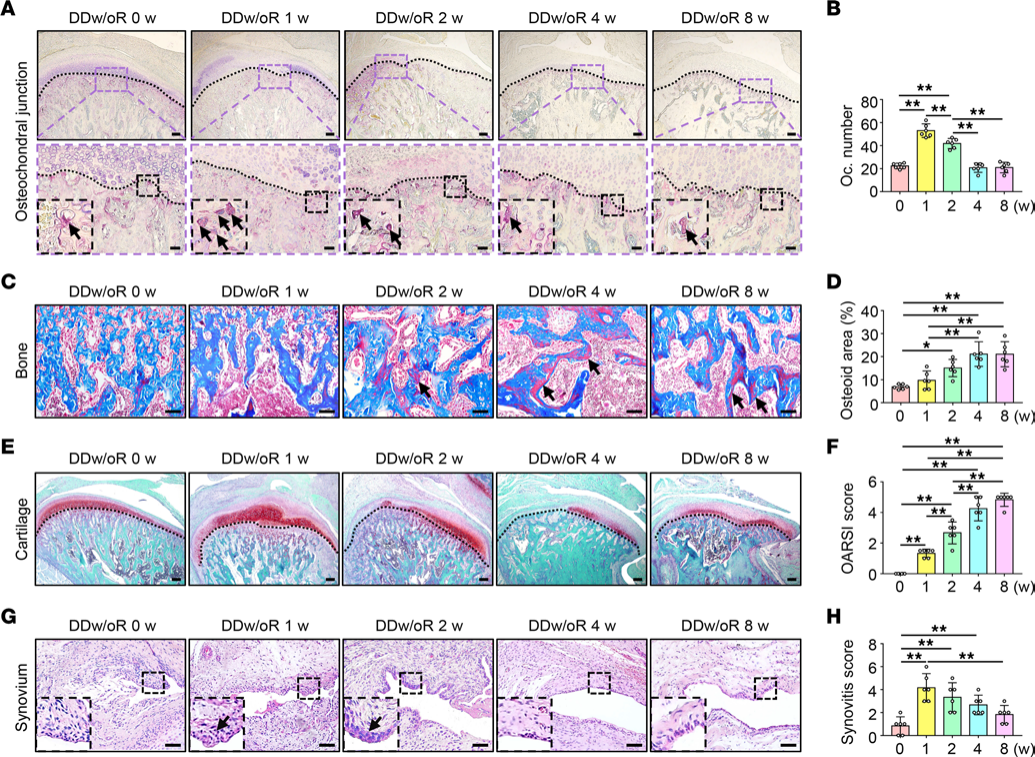
Histopathological changes of TMJs in a rat TMJOA model induced by DDw/oR. Male Sprague-Dawley rats underwent right-sided unilateral DDw/oR surgery. Rats were euthanized at baseline (0 days), 1, 2, 4, and 8 weeks (w) after surgery. (**A**) Representative images of TRAP staining in TMJ sagittal sections. The black dotted line represents the demarcation between articular cartilage and subchondral bone. The black arrows indicate TRAP^+^ osteoclasts with 3 or more nuclei in subchondral bone. Scale bar: (top row) 200 μm, (bottom row) 50 μm. (**B**) Quantitative analysis of the number of TRAP^+^ osteoclasts in subchondral bone of condyles. (**C**) Representative images of Masson’s trichrome staining in TMJ sagittal sections. The black arrows indicate unmineralized bone (osteoid) in subchondral bone. Scale bar: 100 μm. (**D**) Quantitative analysis of the percentage of unmineralized bone (osteoid) area. (**E**) Representative images of Safranin O–fast green staining in TMJ sagittal sections. The black dotted line represents the demarcation between articular cartilage and subchondral bone. Scale bar: 200 μm. (**F**) Quantitative analysis of the OARSI score of articular cartilage. (**G**) Representative images of H&E staining in TMJ sagittal sections. The black arrows indicate synovial lining hyperplasia. Scale bar: 100 μm. (**H**) Quantitative analysis of the synovitis score of TMJ synovium. Data are presented as mean ± 95% CI, and 1 representative image of 6 independent samples per group is shown. Statistical analyses were determined by 1-way ANOVA with Bonferroni’s multiple comparison test. *P < 0.05, **P < 0.01. Abbreviations: Oc, osteoclast; OARSI, Osteoarthritis Research Society International.

The histopathological evaluation also showed that subchondral bone loss was prominent in early-stage TMJOA, and unmineralized bone formation occurred later. The number of TRAP^+^ osteoclasts significantly increased at 1 week after surgery but gradually returned to control levels at 4 weeks after surgery and remained stable until 8 weeks after surgery in the DDw/oR group (Figure 2, A and B). Masson’s trichrome staining showed that the unmineralized bone area was increased starting from 2 weeks and continued at least until 8 weeks (Figure 2, C and D). In addition, the characteristic signs of TMJOA were further evaluated by Safranin O–fast green staining. Proteoglycans in articular cartilage were increased 1 week after surgery but gradually decreased later (Figure 2E). A time-dependent increase in Osteoarthritis Research Society International score occurred after DDw/oR surgery (Figure 2F). Obvious synovial inflammation changes, such as synovial lining hyperplasia, fibrin deposits, and vascularity, were observed at 1 to 2 weeks after surgery in the DDw/oR group (Figure 2G). The synovitis score was significantly increased at 1 week after surgery but returned to control levels at 8 weeks after surgery in the DDw/oR group (Figure 2H). Considering these findings, the specific time points of 1 and 8 weeks after DDw/oR surgery could represent early and late stages of TMJOA, respectively, and were adopted for succeeding animal experiments.

**Figure 3:**
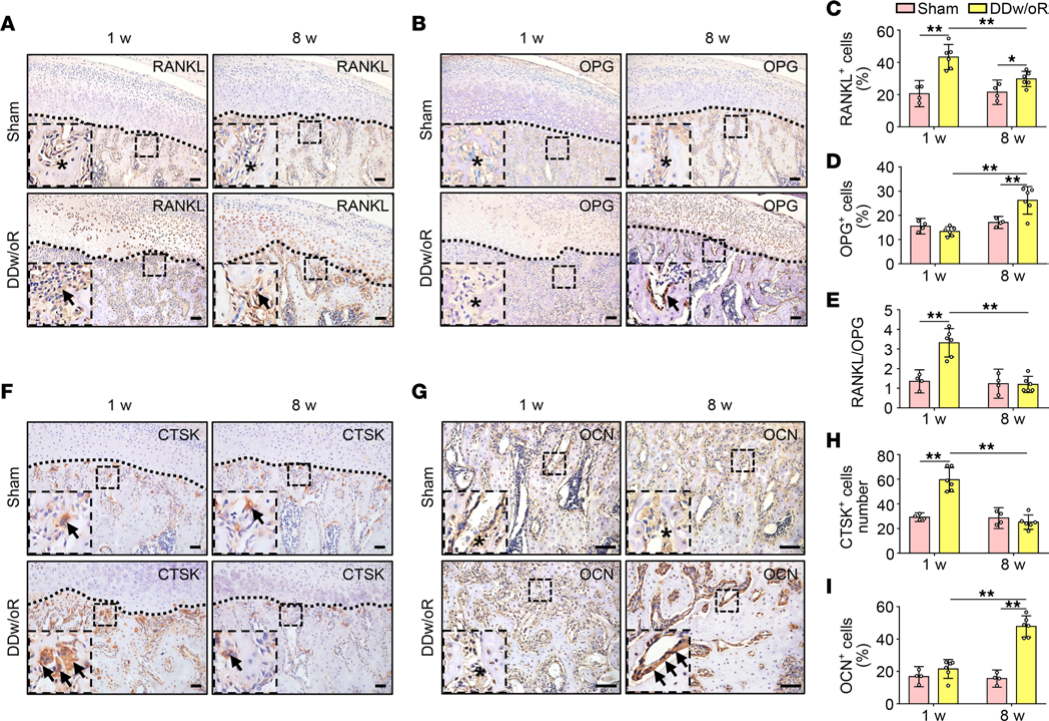
Expression levels of osteoclasts and osteoblasts in a rat TMJOA model induced by DDw/oR. Male Sprague-Dawley rats underwent sham or right-sided unilateral DDw/oR surgery. Rats were euthanized at 1 and 8 weeks (w) after surgery. (**A** and **B**) Representative images of (**A**) RANKL and (**B**) OPG IHC staining in TMJ sagittal sections. The black dotted line represents the demarcation between articular cartilage and subchondral bone. The black arrows indicate positive cells in subchondral bone, and the asterisks indicate negative signals in subchondral bone. Scale bar: 50 μm. (**C** and **D**) Quantitative analysis of the percentage of (**C**) RANKL^+^ cells and (**D**) OPG^+^ cells in subchondral bone of condyles. (**E**) Quantitative analysis of the ratio of RANKL^+^ cells to OPG^+^ cells in subchondral bone of condyles. (**F** and **G**) Representative images of (**F**) CTSK and (**G**) OCN IHC staining in TMJ sagittal sections. The black dotted line represents the demarcation between articular cartilage and subchondral bone. The black arrows indicate positive cells in subchondral bone. Scale bar: 50 μm. (**H**) Quantitative analysis of the number of CTSK^+^ cells in subchondral bone of condyles. (**I**) Quantitative analysis of the percentage of OCN^+^ cells in subchondral bone of condyles. Data are presented as mean ± 95% CI, and 1 representative image of 4 to 6 independent samples per group is shown. Statistical analyses were determined by 2-way ANOVA with Bonferroni’s multiple comparison test. *P < 0.05, **P < 0.01. Abbreviations: Oc, osteoclast; OCN, osteocalcin.

IHC staining and Western blot verified the results reported above. The expression of RANKL and cathepsin K (CTSK), and the ratio of RANKL to osteoprotegerin (OPG) were significantly elevated at 1 week after surgery in the DDw/oR group compared with the sham group (Figure 3, A–F and H, and Supplemental Figure 1; supplemental material available online with this article; https://doi.org/10.1172/jci.insight.158874DS1). Meanwhile, the expression of OPG and osteocalcin were not increased significantly until 8 weeks after surgery in the DDw/oR group (Figure 3, B, D, G, and I). In short, these results indicated that subchondral bone remodeling was dominated by marked bone loss in the recent-onset stage of disc displacement but gradually changed to bone formation thereafter. More importantly, the findings confirmed that the surgical DDw/oR was a suitable in vivo model for investigating subchondral bone loss in early-stage TMJOA.

### RANTES-CCRs-Akt2 axis is activated in early-stage TMJOA.

**Figure 4:**
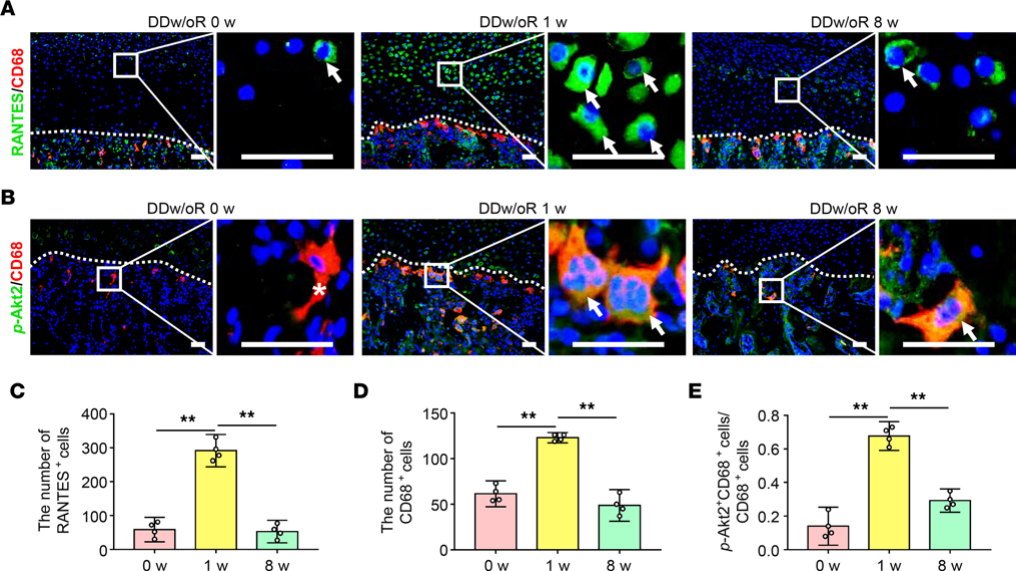
The RANTES-CCRs-Akt2 axis is activated in DDw/oR–induced early-stage TMJOA. Male Sprague-Dawley rats underwent right-sided unilateral DDw/oR surgery. Rats were euthanized at baseline (0 weeks), early (1 week), and late (8 weeks) time points after surgery. (**A**) Representative images of RANTES and CD68 immunofluorescence costaining in TMJ sagittal sections. RANTES^+^ cells appear in green and CD68^+^ cells appear in red. The white dotted line represents the demarcation between articular cartilage and subchondral bone. The white arrows indicate RANTES^+^ cells in articular cartilage. Scale bar: 50 μm. (**B**) Representative images of *p*-Akt2 and CD68 immunofluorescence costaining in TMJ sagittal sections. *p*-Akt2^+^ cells appear in green and CD68^+^ cells appear in red. The white dotted line represents the demarcation between articular cartilage and subchondral bone. The white arrows indicate *p*-Akt2 and CD68 double-positive cells in subchondral bone, and the asterisks indicate CD68 single-positive cells. Scale bar: 50 μm. (**C**) Quantitative analysis of the number of RANTES^+^ cells in articular cartilage of condyles. (**D**) Quantitative analysis of the number of CD68^+^ cells in subchondral bone of condyles. (**E**) Quantitative analysis of the ratio of *p*-Akt2^+^ and CD68 double-positive cells to CD68-positive cells in subchondral bone of condyles. Data are presented as mean ± 95% CI, and 1 representative image of 4 independent samples per group is shown. Statistical analyses were determined by 1-way ANOVA with Bonferroni’s multiple comparison test. **P < 0.01. Abbreviation: w, week.

The role of chemokine RANTES in subchondral bone remodeling in the initiation and/or progression of TMJOA was explored. In the rat model of TMJOA, the number of RANTES^+^ cells, primarily in articular cartilage, significantly increased at 1 week after DDw/oR surgery but returned to the control level at 8 weeks after DDw/oR surgery (Figure 4, A and C). Similarly, the number of macrophages (namely, CD68^+^ cells) residing in the osteochondral junction also increased 1 week after DDw/oR surgery compared with the control (Figure 4, A and D). Findings implied that the increased macrophages in the subchondral bone just beneath articular cartilage were coupled with an increased expression of RANTES.

**Figure 5:**
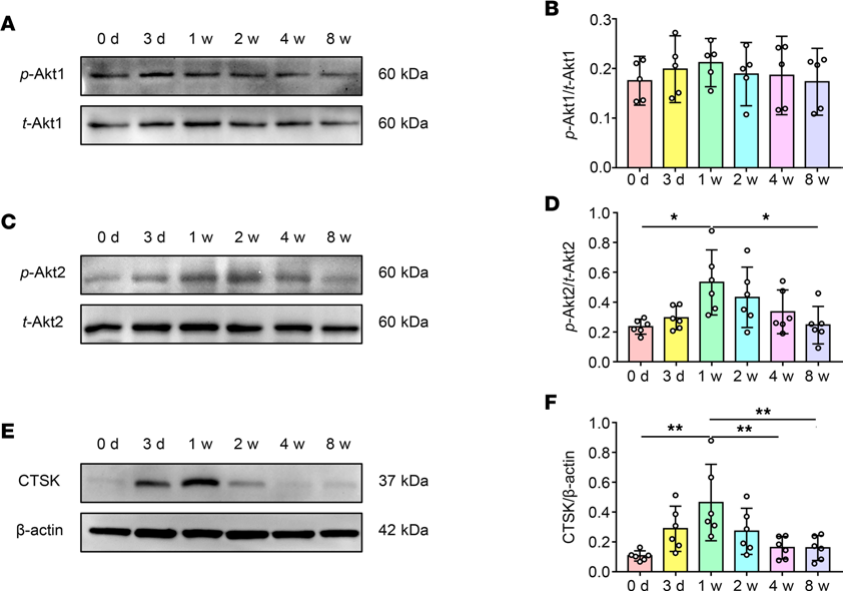
Expression levels of *p*-Akt1, *p*-Akt2, and CTSK in subchondral bone of a rat TMJOA model induced by DDw/oR. Male Sprague-Dawley rats underwent right-sided unilateral DDw/oR surgery. Rats were euthanized at baseline (0 days), 3 days, and 1, 2, 4, and 8 weeks (w) after surgery. (**A**) Representative Western blot bands showing the expression of *p*-Akt1 and *t*-Akt1 in the condylar subchondral bone. (**B**) Quantitative analysis of the relative intensity of *p*-Akt1. The level of *p*-Akt1 is normalized to *t*-Akt1. (**C**) Representative Western blot bands showing the expression of *p*-Akt2 and *t*-Akt2 in the condylar subchondral bone. (**D**) Quantitative analysis of the relative intensity of *p*-Akt2. The level of *p*-Akt2 was normalized to *t*-Akt2. (**E**) Representative Western blot bands showing the expression of CTSK and β-actin in the condylar subchondral bone. (**F**) Quantitative analysis of the relative intensity of CTSK. β-actin was used as an internal control. Data were presented as mean ± 95% CI, and 1 representative image of 5 to 6 independent samples per group is shown. Statistical analyses were determined by 1-way ANOVA with Bonferroni’s multiple comparison test. *P < 0.05, **P < 0.01.

RANTES can recruit immune cells by binding to its CCRs, including CCR1, CCR3, and CCR5. Macrophage recruitment and osteoclast regulation are mediated via CCR5 and Akt pathways (26); therefore, we further investigated the expressions of CCRs and Akt pathways in this model. The expression levels of CCRs were examined and comparable expression levels of CCR1, CCR3, and CCR5 were observed at 0 (baseline), 1, and 8 weeks after DDw/oR surgery (Supplemental Figure 2). Immunofluorescence costaining showed that the percentage of *p*-Akt2^+^&CD68^+^ cells in the CD68^+^ macrophages was significantly increased in subchondral bone at 1 week after DDw/oR surgery but returned to the control level 8 weeks after DDw/oR surgery (Figure 4, B and E). Western blot analysis showed the ratio of *p*-Akt1 to *t*-Akt1 remained relatively constant after DDw/oR surgery at different stages (Figure 5, A and B), but the maximal ratio of *p*-Akt2 to *t*-Akt2 and the maximal expression of CTSK in subchondral bone were observed at 1 week after DDw/oR surgery compared with other time points after surgery (Figure 5, C–F). Taken together, the findings indicated that the RANTES-CCRs-Akt2 axis is activated during subchondral bone loss in early-stage TMJOA.

### RANTES inhibition attenuates subchondral bone loss in early-stage TMJOA.

**Figure 6:**
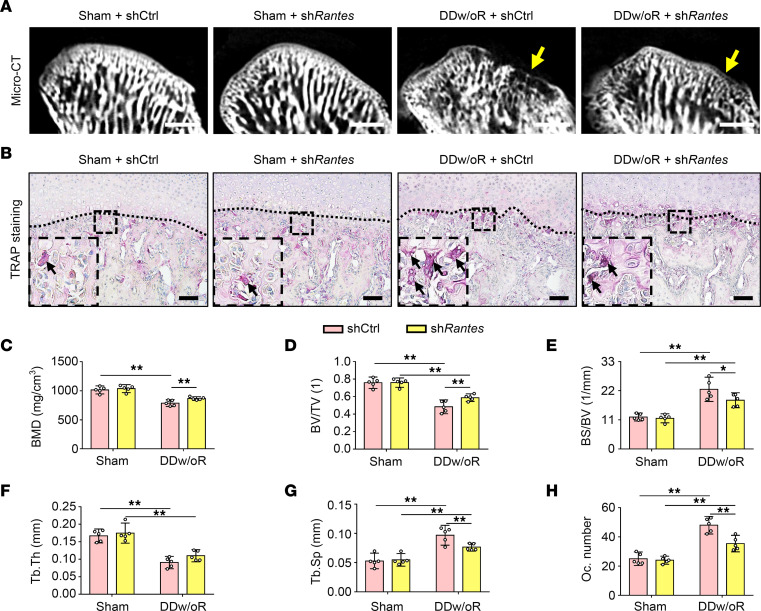
Specific inhibition of RANTES alleviates subchondral bone loss in early-stage TMJOA. Male Sprague-Dawley rats were treated with shCtrl or sh*Rantes* and underwent sham or right-sided unilateral DDw/oR surgery. Rats were euthanized at 1 week after surgery. (**A**) Micro-CT images of TMJ condyles (sagittal view). The yellow arrows show condylar surface erosion. Scale bar: 1 mm. (**B**) Representative images of TRAP staining in TMJ sagittal sections. The black dotted line represents the demarcation between articular cartilage and subchondral bone. The black arrows indicate TRAP^+^ osteoclasts with 3 or more nuclei in subchondral bone. Scale bar: 100 μm. (**C–G**) Quantitative analysis of (**C**) BMD, (**D**) BV/TV, (**E**) BS/BV, (**F**) Tb.Th, and (**G**) Tb.Sp in subchondral bone of TMJ condylar heads determined by micro-CT measurements. (**H**) Quantitative analysis of the number of TRAP^+^ osteoclasts in subchondral bone. Data are presented as mean ± 95% CI, and 1 representative image of 5 independent samples per group is shown. Statistical analyses were determined by 2-way ANOVA with Bonferroni’s multiple comparison test. *P < 0.05, **P < 0.01. Abbreviation: Oc, osteoclast.

To validate the role of RANTES in subchondral bone loss in vivo, adeno-associated virus (AAV) carrying Rantes shRNA (sh*Rantes*) or the vector control (shCtrl) was administered intraarticularly to inhibit the expression of RANTES in articular cartilage in both DDw/oR and sham-operation groups. The knockdown efficiency of sh*Rantes* treatment on DDw/oR rats was 36.7% (Supplemental Figure 3). Subchondral bone loss was partially attenuated in the sh*Rantes*-treated rats compared with the shCtrl-treated group 1 week after DDw/oR surgery (Figure 6A), with higher BMD and BV/TV values and lower values for BS/BV and Tb.Sp (Figure 6, C–G). TRAP^+^ osteoclasts were significantly reduced in the osteochondral junction in the sh*Rantes*-treated rats compared with the shCtrl-treated group 1 week after DDw/oR surgery (Figure 6, B and H). No abnormal subchondral bone remodeling was found in the sham group with either virus or vector control (Figure 6, A–H).

### Akt2 inhibition attenuates subchondral bone loss in early-stage TMJOA.

**Figure 7:**
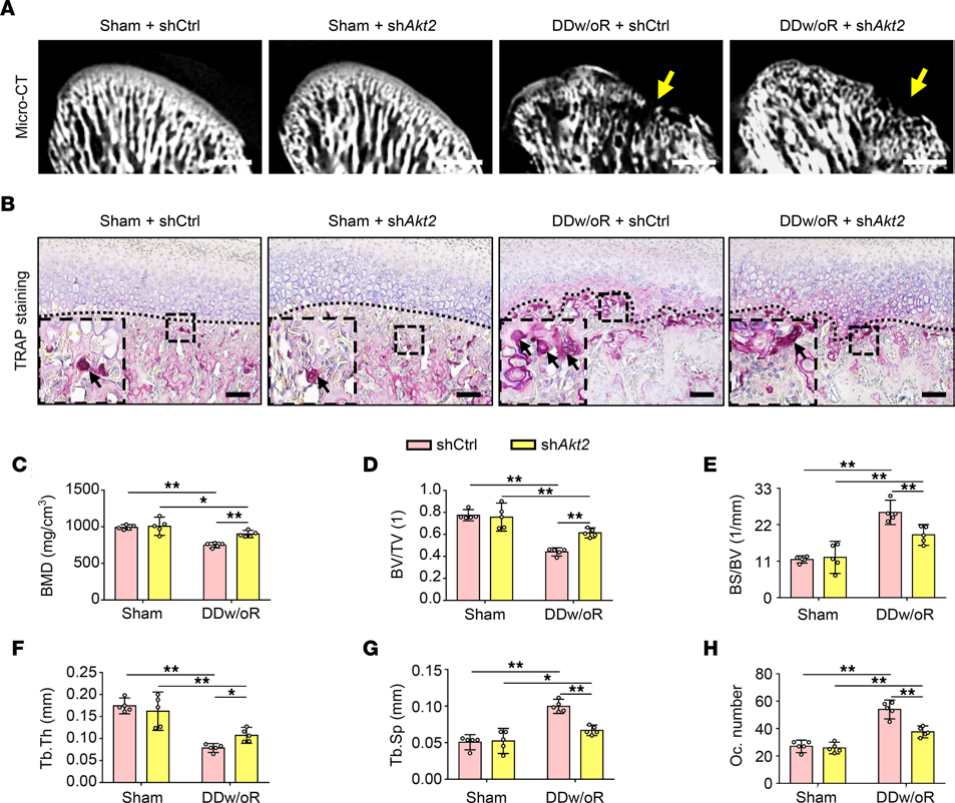
Specific inhibition of Akt2 alleviates subchondral bone loss in early-stage TMJOA. Male Sprague-Dawley rats were treated with shCtrl or sh*Akt2* and underwent sham or right-sided unilateral DDw/oR surgery. Rats were euthanized at 1 week after surgery. (**A**) Micro-CT images of TMJ condyles (sagittal view). The yellow arrows show condylar surface erosion. Scale bar: 1 mm. (**B**) Representative images of TRAP staining in TMJ sagittal sections. The black dotted line represents the demarcation between articular cartilage and subchondral bone. The black arrows indicate TRAP^+^ osteoclasts with 3 or more nuclei in subchondral bone. Scale bar: 100 μm. (**C–G**) Quantitative analysis of (**C**) BMD, (**D**) BV/TV, (**E**) BS/BV, (**F**) Tb.Th, and (**G**) Tb.Sp in subchondral bone of TMJ condylar heads determined by micro-CT measurements. (**H**) Quantitative analysis of the number of TRAP^+^ osteoclasts in subchondral bone. Data were presented as mean ± 95% CI, and 1 representative image of 5 independent samples per group is shown. Statistical analyses were determined by 2-way ANOVA with Bonferroni’s multiple comparison test. *P < 0.05, **P < 0.01. Abbreviation: Oc, osteoclast.

To further validate the role of the Akt2 pathway in subchondral bone loss in vivo, sh*Akt2* was administered i.v. to inhibit the expression of Akt2 in subchondral bone in both DDw/oR and sham-operation groups. Similar results were observed in the sh*Akt2*-treated groups. The knockdown efficiency of sh*Akt2* treatment on DDw/oR rats was 61.8% (Supplemental Figure 4). The micro-CT results demonstrated that almost all the parameters of subchondral bone loss were significantly rescued in the sh*Akt2*-treated group compared with the shCtrl-treated group after DDw/oR surgery (Figure 7, A and C–G). Tartrate-resistant acid phosphatase (TRAP) staining also showed that TRAP^+^ osteoclasts were reduced in the osteochondral junction in the sh*Akt2*-treated rats compared with the shCtrl-treated group 1 week after DDw/oR surgery (Figure 7, B and H). No significant difference was observed in the sham-operation group with either virus or vector control (Figure 7, A–H).

### Increased joint loading results from TMJ disc displacement.

**Figure 8:**
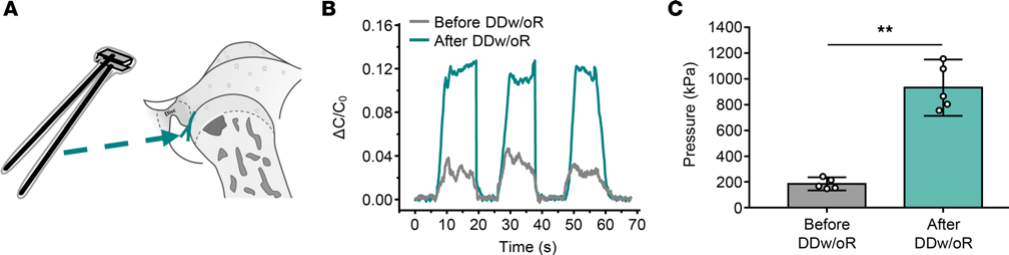
Increased pressure on the anterior surface of condyles is yielded by TMJ disc displacement. Male Sprague-Dawley rats underwent right-sided unilateral DDw/oR surgery. (**A**) The graphic illustration of capacitance-based force-sensing system fastened to the anterior surface of condyles. (**B**) Capacitance variations (ΔC) during the cycles from mouth closure to mouth opening in a rat before and after DDw/oR surgery. C_0_, initial capacitance. (**C**) The pressures on the anterior surface of condyles at the maximum passive mouth-opening position before and after DDw/oR surgery. Data are presented as mean ± 95% CI, and 1 representative image of 5 independent samples per group is shown. Statistical analysis was determined by Student’s *t*-test. **P < 0.01.

To verify the consequence of disc displacement on joint loading, a capacitance-based force-sensing system was developed to measure the articular surface pressure before and after disc displacement in vivo (Figure 8A). Capacitance variations were stable during the 3 passive mouth-opening and mouth-closing cycles both before and after DDw/oR surgery, and the variations were significantly elevated at the maximum passive mouth-opening position after DDw/oR surgery (Figure 8B). Based on the pressure-capacitance sensing curves, the mean pressure over the sensor area in contact with the anterior surface of condyles generated by disc displacement (931.72 ± 56.50 kPa) was approximately 5 times higher than when the articular disc was in the normal position (184.68 ± 54.60 kPa) (Figure 8C).

### RANTES-CCRs-Akt2 axis is activated by increased joint loading.

**Figure 9:**
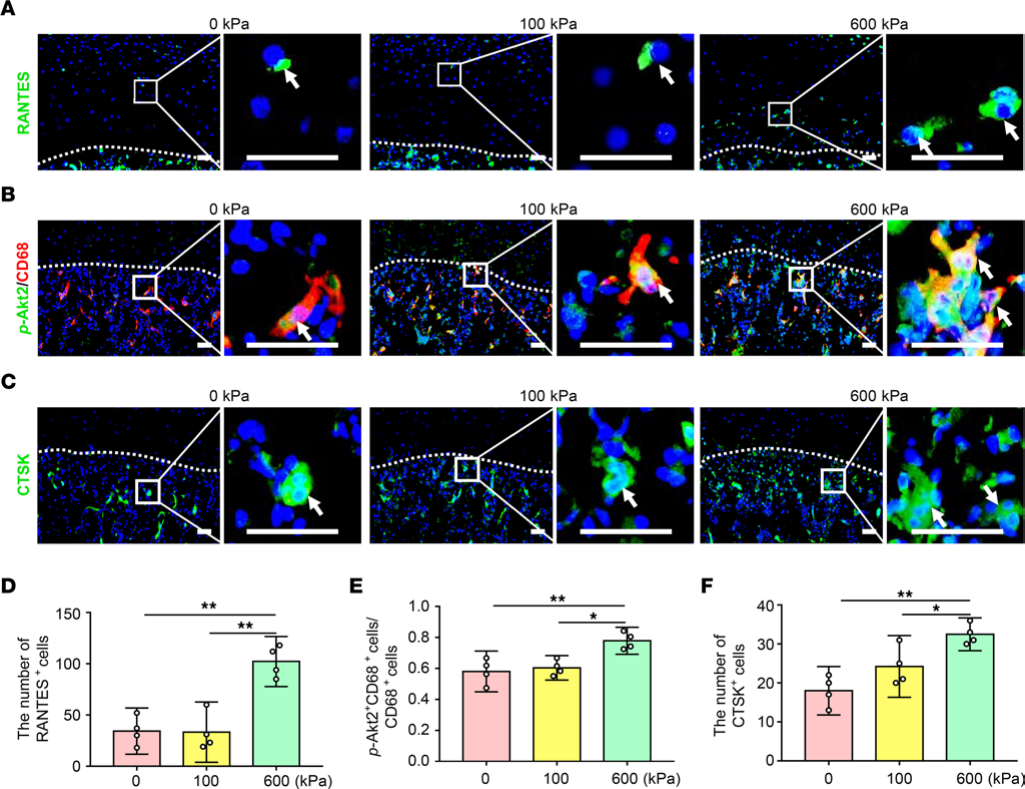
The RANTES-CCRs-Akt2 axis is activated by increased pressure. Isolated rat condylar heads were stimulated under 0, 100, and 600 kPa in vitro. (**A**) Representative images of RANTES immunofluorescence staining in condylar sagittal sections. The white dotted line represents the demarcation between articular cartilage and subchondral bone. The white arrows indicate RANTES^+^ cells in articular cartilage. Scale bar: 50 μm. (**B**) Representative images of *p*-Akt2 and CD68 immunofluorescence costaining in condylar sagittal sections. *p*-Akt2^+^ cells appear in green and CD68^+^ cells appear in red. The white dotted line represents the demarcation between articular cartilage and subchondral bone. The white arrows indicate *p*-Akt2 and CD68 double-positive cells in subchondral bone. Scale bar: 50 μm. (**C**) Representative images of CTSK immunofluorescence staining in condylar sagittal sections. The white dotted line represents the demarcation between articular cartilage and subchondral bone. The white arrows indicate CTSK^+^ cells in subchondral bone. Scale bar: 50 μm. (**D**) Quantitative analysis of the number of RANTES^+^ cells in articular cartilage. (**E**) Quantitative analysis of the ratio of *p*-Akt2^+^ and CD68 double-positive cells to CD68-positive cells in subchondral bone. (**F**) Quantitative analysis of the number of CTSK^+^ cells in subchondral bone. Data are presented as mean ± 95% CI, and 1 representative image of 4 independent samples per group is shown. Statistical analyses were determined by 1-way ANOVA with Bonferroni’s multiple comparison test. *P < 0.05, **P < 0.01.

To prove that increased joint loading activates the RANTES-CCRs-Akt2 axis, pressures of 100 kPa and 600 kPa were applied to the isolated rat condylar heads in vitro to simulate loading on the anterior surface of condyles in normal disc position and DDw/oR, respectively. Immunofluorescence staining showed that the number of RANTES^+^ cells in articular cartilage significantly increased under 600 kPa (Figure 9, A and D). The percentage of p-Akt2^+^/CD68^+^ cells in CD68^+^ macrophages, and the number of osteoclasts (namely, CTSK^+^ cells) in subchondral bone also increased under 600 kPa compared with the 0 or 100 kPa groups (Figure 9, B, C, E, and F). Collectively, findings revealed that increased loading on the anterior surface of condyles could induce activation of the RANTES-CCRs-Akt2 axis and increase osteoclast formation.

### RANTES-CCRs-Akt2 axis activation facilitates osteoclast formation.

**Figure 10:**
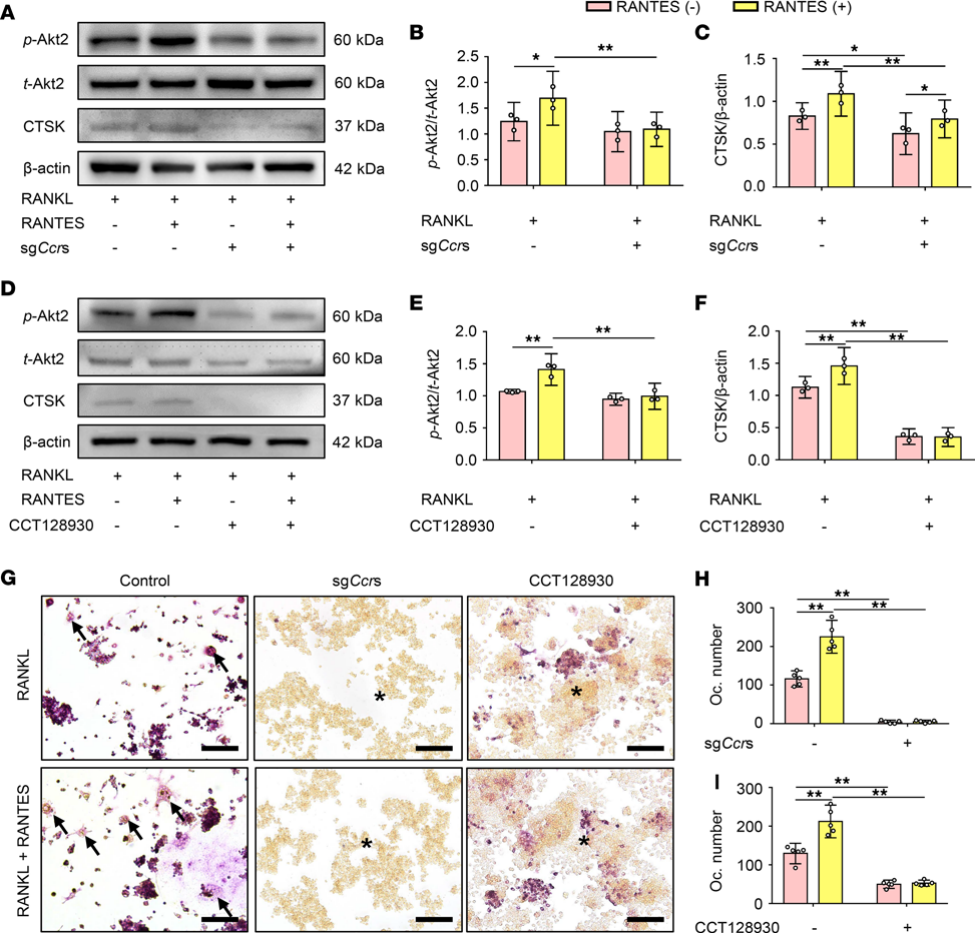
The RANTES-CCRs-Akt2 axis facilitates osteoclast formation. RAW264.7 cells were treated with different osteoclast-inducing conditions in vitro. (**A**) Representative Western blot bands of the expression of *p*-Akt2, *t*-Akt2, CTSK, and β-actin. (**B** and **C**) Quantitative analysis of the relative intensity of (**B**) *p*-Akt2 and (**C**) CTSK. The level of *p*-Akt2 was normalized to *t*-Akt2. The level of CTSK was normalized to β-actin. (**D**) Representative Western blot bands of the expression of *p*-Akt2, *t*-Akt2, CTSK, and β-actin. (**E** and **F**) Quantitative analysis of the relative intensity of (**E**) *p*-Akt2 and (**F**) CTSK. The level of *p*-Akt2 was normalized to *t*-Akt2. The level of CTSK was normalized to β-actin. (**G**) Representative images of TRAP staining. The black arrows indicate TRAP^+^ osteoclasts, and the asterisks indicate negative signals. Scale bar: 100 μm. (**H**) Quantitative analysis of the number of TRAP^+^ osteoclasts. (**I**) Quantitative analysis of the number of TRAP^+^ osteoclasts. Data are presented as mean ± 95% CI, and 1 representative image of 3 to 5 independent experiments with biological replicates is shown. Statistical analyses were determined by 2-way ANOVA with Bonferroni’s multiple comparison test. *P < 0.05, **P < 0.01. Abbreviation: Oc, osteoclast.

To better understand the function of the RANTES-CCRs-Akt2 axis during subchondral bone loss, RANTES activation of the Akt2 pathway for regulating osteoclast formation was explored using a macrophage cell line. Real-time quantitative PCR (RT-qPCR) revealed that RANTES stimulation markedly increased the expression of osteoclast-associated genes, including *Trap*, *Ctsk*, and *Mmp9*, especially in the 100 ng/mL group (Supplemental Figure 5). Moreover, RANTES stimulation increased the ratio of *p*-Akt2 to *t*-Akt2 and the expression of CTSK (Figure 10, A–F). TRAP staining showed that RANTES promoted osteoclast formation in the presence of RANKL (Figure 10, G–I). The findings suggested that RANTES could upregulate *p*-Akt2 expression in macrophages and facilitate osteoclast formation.

To authenticate that RANTES regulates *p*-Akt2 expression through CCRs, the genes of RANTES receptors, including *Ccr1*, *Ccr3*, and *Ccr5*, were knocked out via sgRNAs. The knockdown efficiency of sg*Ccr*1, sg*Ccr*3, and sg*Ccr*5 were 54.8%, 64.8%, and 50.1%, respectively (Supplemental Figure 6). Under an osteoclast-inducing condition, sg*Ccr*s (see Methods) significantly decreased the expression of *p*-Akt2 and CTSK (Figure 10, A–C). Moreover, the activation of Akt2 was inhibited by the Akt2 inhibitor CCT128930, which further resulted in decreased expression of *p*-AKT2 and CTSK (Figure 10, D–F). These treatments inhibited osteoclast formation in the sg*Ccr*s-treated groups and the CCT128930-treated groups regardless of the presence or absence of RANTES (Figure 10, G–I).

### RANTES as a potential biomarker of early-stage TMJOA in humans.

**Figure 11:**
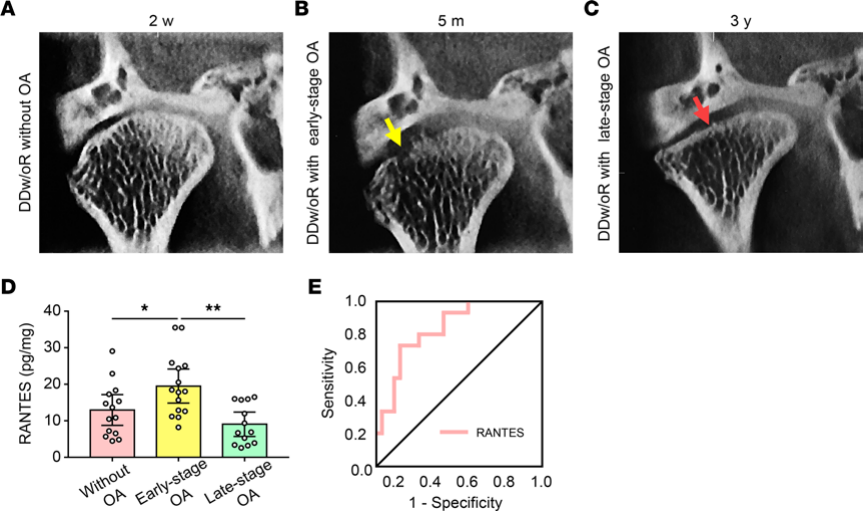
The relationship between the concentration of RANTES and different stages of TMJOA in humans. Synovial fluid samples were collected from patients diagnosed with TMJ DDw/oR. (**A–C**) Radiographic changes of TMJ condylar bone remodeling (coronal view) in the natural course of a patient with DDw/oR. (A) Normal condylar bone with no sign of OA 2 weeks (w) after disc displacement. (**B**) Early-stage OA after 5 months (m). (**C**) Late-stage OA after 3 years (y). The yellow arrow shows condylar bone erosion. The red arrow indicates condylar bone flattening and sclerosis. (**D**) Comparison of RANTES levels in synovial fluid of patients with different stages of DDw/oR. (**E**) ROC curve of the logistic regression model of RANTES drawn by the back-substitution method. The AUC of RANTES was 0.798. Data are presented as mean ± 95% CI, and 1 representative of 13 to 15 independent samples per group is shown. Statistical analysis was determined by 1-way ANOVA with Bonferroni’s multiple comparison test. *P < 0.05, **P < 0.01.

Given the aforementioned findings, the relationship between the concentration of RANTES and different stages of TMJOA was investigated to validate the role of RANTES as a potential biomarker for early-stage TMJOA. Bone phenotypes based on CBCT images (Figure 11, A–C) and RANTES concentrations in the TMJ synovial fluids of 42 patients diagnosed with DDw/oR were analyzed. The level of RANTES in synovial fluid of patients with DDw/oR with early-stage TMJOA was significantly higher than in that of patients without TMJOA and late-stage TMJOA (Figure 11D). Logistic regression modeling and receiver operating characteristic (ROC) curve indicated that RANTES was associated with subchondral bone loss in early-stage TMJOA. The AUC of RANTES was 0.798 (Figure 11E), indicating that RANTES is a potential biomarker with satisfactory diagnostic accuracy in distinguishing DDw/oR in patients with early-stage OA from other patients with DDw/oR.

## Discussion

This study was undertaken to determine if increased joint loading secondary to disc displacement produces subchondral bone loss and to characterize the underlying mechanisms for early-stage TMJOA. By means of the capacitance-based force-sensing system and in vivo and in vitro testing, DDw/oR was found to yield significantly higher pressure on the anterior surface of condyles. The increased joint loading activated the RANTES-CCRs-Akt2 axis and promoted osteoclast formation, leading to subchondral bone loss. Furthermore, inhibition of RANTES or Akt2 attenuated subchondral bone loss and resulted in improved subchondral bone microstructure. The clinical study indicated that increased RANTES was associated with early-stage TMJOA after disc displacement and could be a potential biomarker. Considering the in vivo, in vitro, and clinical evidence, all research hypotheses were supported and the RANTES-CCRs-Akt2 axis could be targeted for treating early-stage TMJOA.

In the rat model we used, subchondral bone loss occurs 3 to 7 days after disc displacement. This phenomenon further supports the view that joint deterioration is characterized by subchondral bone loss in early-stage TMJOA (7, 8). In addition, the surgery-induced DDw/oR model mimicked radiographic manifestations of early-stage TMJOA associated with recent-onset DDw/oR in patients, including condylar cortex discontinuity and condylar surface erosion (21). These findings suggest that the rat model induced by DDw/oR is valid for exploring subchondral bone loss in early-stage TMJOA.

Mechanical joint overloading is considered an important factor for subchondral bone loss and promoting TMJOA (17, 18). Although some studies on the biomechanics of TMJs have been performed in vitro (27, 28) and in vivo (29), articular surface loading with DDw/oR has not been often investigated. The capacitance-based force sensor comprises polydimethylsiloxane (PDMS) and silver nanowire (AgNW) film. PDMS possesses good biocompatibility as well as flexibility and could be adapted to fit the shape of the articular surface. Moreover, the AgNW film has excellent electrical conductivity and mechanical ductility, which contributed to the consistency of the capacitance signal output when the PDMS sensors were repeatedly loaded and unloaded (30, 31). Given its reliability and ability to measure loads on irregular surfaces in small cavities, the force-sensing system was a useful tool for in vivo studies (32). Pressure on the anterior surface of condyles after disc displacement reached 931.72 ± 56.50 kPa, which was approximately 5 times higher than that before disc displacement (184.68 ± 54.60 kPa). The pressure measured directly on the anterior surface of condyles when discs were displaced without reduction corroborated a previous study using finite-element analysis (33) and indicated that increased mechanical loading on the anterior surface of condyles could be caused by disc displacement.

Previous studies have shown that inflammatory mediators triggered by mechanical loading are involved in OA (34–38). Although RANTES was increased in synovial fluids of patients with TMJOA (23), it was unknown whether increased joint loading directly induced the upregulation of RANTES and contributed to early-stage TMJOA. RANTES, an inflammatory chemokine that has receptors, including CCR1, CCR3, and CCR5, can recruit immune cells to inflammatory sites and is involved in autoimmune diseases (39, 40). Emerging clinical evidence indicated that the expression of RANTES is elevated in several diseases involving bone loss, such as rheumatoid arthritis, fatty oxide osteoporosis and osteolysis, and cancer-associated bone destruction (41–43). Some studies have reported that transgenic mice with *Ccr1/Ccr5* gene KO could attenuate osteoclast differentiation (26, 44, 45). The present study determined that the expression of RANTES was increased substantially in articular cartilage when the TMJs were loaded both in vitro and in vivo. Concurrently, the number of macrophages and osteoclasts increased at the osteochondral junction in early-stage TMJOA, which suggested that RANTES in articular cartilage could recruit macrophages to the osteochondral junction and promote osteoclast formation. Findings further confirmed that RANTES was increased only in early-stage TMJOA and could be a potential biomarker for subchondral bone loss in early-stage TMJOA in humans.

Although accumulating literature has demonstrated that RANTES could recruit and repolarize macrophages by binding to its receptors and activating the downstream pathway involving PI3, Akt, and MAP kinases (46–48), in this study, only *p*-Akt2, rather than *p*-Akt1 or other downstream molecules, was upregulated in macrophages in the subchondral bone. Previous studies indicated that activation of Akt2 might regulate macrophage M1 polarization and augment the inflammatory response (49–51), but little is known about the effect of Akt2 on osteoclast formation. In this study, RANTES activated the Akt2 pathway and promoted osteoclast formation. The inhibition of CCRs or Akt2 expression could significantly decrease the ratio of *p*-Akt2 to *t*-Akt2 and the number of osteoclasts in vitro. This implied that CCRs and Akt2 might be key molecules contributing to osteoclast formation. Genetic inhibition of RANTES or Akt2 significantly decreased osteoclast formation and alleviated subchondral bone loss in early-stage TMJOA in vivo. Collectively, the data indicated that RANTES in articular cartilage could induce osteoclast formation in subchondral bone via binding to CCRs and activating the Akt2 pathway, and inhibition of the RANTES-CCRS-Akt2 axis could attenuate subchondral bone loss and improve subchondral bone microstructure in TMJOA development.

In conclusion, this study demonstrated that increased joint loading secondary to disc displacement mediated subchondral bone loss; specifically, the expression of RANTES in articular cartilage is increased, resulting in recruitment of macrophages in the subchondral bone to the osteochondral junction and promoting osteoclast formation by activating the Akt2 pathway. Our results support that the RANTES-CCRs-Akt2 axis has important regulatory functions in subchondral bone remodeling, and the results provide further knowledge about the crucial role of increased joint loading secondary to disc displacement in the development of TMJOA.

## Methods

### In vivo animal model

#### Rat model of TMJOA.

Six-week-old male Sprague-Dawley rats (180–200 g) were purchased from Vital River Laboratory, fed in a specific pathogen-free environment for 2 weeks before operations, and maintained at room temperature in a 12-hour light/dark cycle with easy access to water and food.

Rats were anesthetized with 1% sodium pentobarbital and randomized to the DDw/oR and sham-operated groups. Surgically unilateral DDw/oR was produced by modifying the procedures used by Togni et al. (52) to induce TMJOA, while the unilateral sham operation was carried out on independent rats under anesthetized conditions. In the DDw/oR group, a needle with a 5-0 suture was passed vertically through the posterior band of the disc and anchored to the bend point of the zygomatic arch. The disc was thus stretched anteriorly, causing the whole disc to be positioned in front of the condyle (Supplemental Figure 7). The sham group was operated on similarly but without displacing the disc. The rats were sacrificed at 0 days, 3 days, and 1, 2, 4, and 8 weeks after DDw/oR and sham operations.

#### AAV infection.

Two rescue therapies were applied to intervene with the DDw/oR-induced subchondral bone loss in the rat model. In vivo gene knockdown was achieved by AAV. Construction and production of recombinant AAV serotype 9 carrying *Rantes* shRNA (sh*Rantes*) or shCtrl with a COL2A1 promoter coupled to an EGFP tag were manufactured by GeneChem Company. The vector was COL2A1p-EGFP-MCS-SV40 PolyA. AAV9 containing *Akt2* shRNA (sh*Akt2*) or shCtrl linked with an EGFP tag was completed by GeneChem using the U6-MCS-CAG-EGFP vector.

To specifically inhibit the expression of RANTES in articular cartilage of condyle, sh*Rantes* (or shCtrl) was injected intraarticularly at a final titer of 2 × 10^12^ transducing units/mL (total volume of 50 μL/rat) on the 10th day before surgery. To inhibit the expression of Akt2 in subchondral bone, sh*Akt2* (or shCtrl) was injected i.v. at a final titer of 1 × 10^12^ transducing units/mL (total volume of 100 μL/rat) 10 days before the DDw/oR or sham operations. All rats were euthanized and assessed 1 week after DDw/oR and sham operations, and the transfection efficiency of AAV was routinely checked under fluorescence microscopy (Supplemental Figures 3 and 4).

#### Articular surface-pressure measurement.

To verify the consequence of disc displacement on joint loading in vivo, a capacitance-based force-sensing system was developed and modified to measure the pressure on the anterior surface of condyles (53). PDMS forms the core sensory element for the sensing device, and AgNW film electrodes with copper tape wires adhered to the upper and lower surfaces of PDMS. Considering the shape of the articular surface and the space of the lower compartment of the TMJ cavity, the size of the sensor was designed to be 1.0 mm long, 2.0 mm wide, and 0.6 mm thick to ensure it could detect the pressure on the anterior surface of condyles without interfering with jaw movements.

To monitor the loading on the anterior surface of condyles, the sensing device was sterilized, positioned, and fastened onto the anterior portion of the articular surface by medical gum (Compant) before incision closure (Supplemental Figure 8A). The pressure over the sensor area in contact with the anterior surface of condyles was measured before and after disc displacement.

Capacitance variations were monitored during passive jaw opening and closing in a rat with the normal disc position under anesthesia. The articular disc was surgically displaced in the same rat and the pressure on the anterior surface of condyles was measured again during passive mouth opening and closing. Jaw opening before and after disc displacement was standardized at 20 mm (54), and capacitance variations on the anterior surface of condyles were recorded (Supplemental Figure 8B).

To transform capacitance variations to relative pressure values, the force-capacitance sensing curves obtained (Supplemental Figure 8C) were calibrated against a force-measuring apparatus comprising a high-precision universal testing machine, a Mark-10 Force Gauge to record pressure stimulus, an LCR meter (IM3536, HIOKI, Japan) to deliver AC voltage (1 V, 1 MHz), and software for recording real-time capacitance signals. The pressure values on the anterior surface of condyles were calculated according to the pressure-capacitance sensing curves.

#### Micro-CT analysis.

Rats from the DDw/oR and sham-operation groups were anesthetized and then sacrificed. The TMJs were removed and fixed in 10% formalin overnight. Images of condyles were scanned under 80 kV, 500 μA, and 33.658 μm pixel size by an Inveon micro-CT system (Siemens). Radiographs were reconstructed and analyzed with Inveon Research Workplace software. The region of interest covered the condylar head and a total of 70 consecutive cross-sectional images from the most superior point of the condylar head were used. The following parameters, including BMD, the BV/TV, the BS/BV, Tb.Th, trabecular number, Tb.Sp, and the length, width, and height of the condylar head (Supplemental Figure 9) were measured and compared between the DDw/oR and sham groups at 0 days, 3 days, and 1, 2, 4, and 8 weeks after surgery.

#### Histopathological evaluations.

The TMJs from both the DDw/oR and sham-operation groups were removed, fixed in 10% formalin, decalcified in 10% EDTA (pH 7.4), and embedded in paraffin. The sagittal sections of the TMJs (5 μm thick) were processed for staining. TRAP staining (Sigma‑Aldrich), Masson’s trichrome staining, Safranin O–fast green staining, and H&E staining (Solarbio) were performed according to the manufacturers’ manuals. A modified Osteoarthritis Research Society International scoring system was used to evaluate the articular cartilage changes in rats (Supplemental Table 1) (55, 56). A synovitis scoring system was used to observe synovial pathologies, which contained 4 criteria: synovial lining hyperplasia, inflammatory infiltrate, fibrin deposits, and vascularity (Supplemental Table 2) (57, 58).

Immunostaining was performed following standard protocols. The primary Abs included CTSK (1:200; Abcam, ab19027), osteocalcin (1:100; R&D, MAB1419-SP), RANKL (1:100; Abcam, ab62516), OPG (1:100; ABclonal, A2100), RANTES (1:20; Invitrogen, Thermo Fisher Scientific, 710001), CCR1 (1:100; ABclonal, A18341), CCR3 (1:100; Abcam; ab32512), CCR5 (1:100; Abnova, PAB18117), *p*-Akt2 (1:100; Bioss, bs-4089R), and CD68 (1:400; Bio-Rad, MCA341GA). Then, HRP-streptavidin detection system (Zsbio) or fluorescence-conjugated secondary Ab (Jackson ImmunoResearch) was used to detect immunoactivity. Hematoxylin or DAPI was used for nuclear staining.

Images were captured with a light microscope (Olympus). Quantitative analyses were performed in a blinded fashion with ImageJ software (Media Cybernetics Inc.).

#### RT-qPCR.

Total cellular RNA was extracted by TRIzol (Invitrogen), and the concentrations were measured with a NanoDrop 8000 spectrophotometer (Thermo Fisher Scientific). PrimeScript RT Master Mix (TAKARA, RR036A) was used to generate single-stranded cDNA. A 7500 Real-Time PCR System (Thermo Fisher Scientific) with SYBR Green reagent (Roche Diagnostics) was used for RT-qPCR. Osteoclast-associated genes, including *Trap*, *Ctsk*, and *Mmp9*, were detected. The sequences of target-specific primers are listed in Supplemental Table 3.

#### Western blot.

Subchondral bone tissues or RAW 264.7 cells were lysed in RIPA buffer (Solarbio) supplemented with PMSF, a phosphatase inhibitor, and protease inhibitor (Sigma-Aldrich). The lysates were centrifuged at 4°C, 12,000*g* for 20 minutes. Total protein concentration was measured by bicinchoninic acid Protein Assay Kit (Thermo Scientific). The aliquot of proteins was subjected to SDS-PAGE and transferred electrophoretically to polyvinylidene fluoride membranes (Millipore). After blocking with 5% nonfat milk, the membranes were incubated with primary Ab, including *p*-Akt1 (1:1000; Cell Signaling Technology, 9018), Akt1 (1:1000; Cell Signaling Technology, 2938), *p*-Akt2 (1:1000, Cell Signaling Technology, 8599), Akt2 (1:1000, Cell Signaling Technology, 3063), CTSK (1:1000; Abcam, ab19027), RANKL (1:1000; Abcam, ab62516), OPG (1:1000; ABclonal, A13250), and β-actin (1:10000; Proteintech, 66009-1-lg). After washing, the blots were probed with HRP-conjugated secondary Ab (1:10,000; Cell Signaling Technology) and subjected to enhanced chemiluminescence detection (Thermo Fisher Scientific). The intensity of bands was quantified by ImageJ and normalized to the density of the internal control.

### In vitro culture models

#### Pressurized tissue culture.

The numerically controlled loading apparatus was developed by Naturethink Company (NK-700, Naturethink Life Technology Ltd.). Condylar heads (2.0 mm high, with cartilage and subchondral bone) obtained from 8-week-old rats were put into 96-well plates with DMEM (Gibco) containing 10% FBS (Gibco) and 1% penicillin–streptomycin (Gibco) and set into a cylindrical chamber at 37°C with 5% CO_2_ atmosphere. A numerically controlled apparatus was used to apply 100 or 600 kPa pressure on isolated rat condylar heads in sealed chambers for 24 hours. The controls were put into the same chambers and cultured without pressure. The samples were then processed for histological evaluation.

#### Cell culture model.

The RAW 264.7 murine macrophage cells (Peking Union Medical College) were cultured in DMEM supplemented with 10% FBS and 1% penicillin–streptomycin at 37°C with 5% CO_2_ atmosphere. The medium was changed every 2 days. To induce osteoclast formation, RAW 264.7 cells were induced with 5 ng/mL RANKL (R&D Systems) and 100 ng/mL RANTES (R&D Systems). After 4 days, cells were fixed in 4% paraformaldehyde and then subjected to TRAP staining. TRAP-positive multinuclear (3 or more nuclei) cells were deemed osteoclasts. For each well, 9 random fields were captured and counted using a light microscope. Data were acquired from 3 to 5 independent experiments with biological replicates.

#### Lentivirus infection and drug administration.

In vitro gene KO was achieved by lentivirus vectors. sgRNAs specifically targeting *Ccr1*, *Ccr3*, or *Ccr5* were designed by GeneChem. The recombinant lentivirus vectors carrying sg*Ccr1*, sg*Ccr3*, and sg*Ccr5* linked with a puromycin tag were purchased from GeneChem and used for RAW 264.7 cells infection at an MOI of 20. For viral infection, RAW 264.7 cells were infected with lentivirus plus 4 μg/mL polybrene (Sigma-Aldrich) for 8 hours and then cultured with an ordinary complete medium. At 96 hours after infection, 10 μg/mL puromycin (Sigma-Aldrich) was added to select the infected cells. The transfection efficiency was checked via Western blot (Supplemental Figure 6). The RAW 264.7 cell line with the KO of *Ccr*s was screened with puromycin and termed sg*Ccr*s, and an empty lentivirus was applied as a negative control. After lentivirus infection, cells were stimulated by RANKL and RANTES for the next assay.

For the inhibition of Akt2 activity in RAW 264.7 cells, the Akt2 inhibitor CCT128930 (10 μM; Selleck) or an equivalent volume of DMSO was added into the medium for 4 days under an osteoclast-inducing condition.

### Human study

#### Human participants.

Forty-two synovial fluid samples were collected from patients diagnosed with TMJ DDw/oR who were seeking treatment. Patient inclusion criteria were based on the Diagnostic Criteria for Temporomandibular Disorders (59). The patients were further divided into 3 subgroups according to CBCT findings (Supplemental Table 4): DDw/oR without OA (*n* = 14), DDw/oR with early-stage OA (*n* = 15), and DDw/oR with late-stage OA (*n* = 13). Radiographic signs of discontinuity of the articular cortex and/or surface erosion or destruction of the condyle were considered early-stage OA changes, whereas deviation in form, sclerosis, osteophyte formation, and cyst-like lesions were deemed late-stage OA (60). Patient exclusion criteria were as follows: (a) history of prior temporomandibular disorders therapy; (b) history of joint trauma; and (c) presence of systematic joint diseases (e.g., rheumatoid arthritis).

#### Synovial fluid collection.

Synovial fluid samples were collected during TMJ arthrocentesis. Saline solution (1.0 mL) was injected into the upper compartment of the TMJ cavity and mixed with synovial fluid 3 times by repeated aspiration and re-injection. Afterward, 1.0 mL of synovial fluid was collected before any treatment. Samples contaminated with blood were discarded. All samples were centrifuged at 1500*g* at 4°C for 10 minutes to remove cells and tissue debris, stored in aliquots at -80°C, and analyzed with the following assays.

#### ELISA.

For synovial fluid samples, total protein concentration was used as the standard for normalizing cytokine levels in the different samples, which was performed by the bicinchoninic acid assay method. Chemokine RANTES was evaluated using the Human RANTES ELISA Kit (R&D Systems). The sensitivity of the assay was 2.0 pg/mL for RANTES. The measurements were carried out using freshly thawed aliquots of synovial fluid samples in duplicate.

### Statistics

Continuous data are presented as mean ± 95% CI. The normality of a population was assessed by the Shapiro-Wilk test. For normally distributed variables, a 2-tailed Student’s *t* test was used for 2-group comparison, 1-way ANOVA with Bonferroni’s multiple comparison test was used for multigroup data sets, and 2-way ANOVA with Bonferroni’s multiple comparison test was used for serial comparison of the 2 × 2 experimental designs. For nonnormally distributed variables, Kruskal-Wallis nonparametric test was chosen for analysis. The logistic regression model and ROC curve were chosen to quantitatively evaluate the diagnostic accuracy. Statistical analyses were performed using SPSS 21.0 software (IBM) with a significance level of *P* < 0.05.

### Study approval

Approval for the animal research was obtained from the Peking University Animal Ethics Committee (LA2019310). The human study was approved by the Biomedical IRB of the Peking University School and Hospital of Stomatology (PKUSSIRB-201947100), and written informed consent was attained prior to participation from all participants.

## Author contributions

SYF, JL, RRW, YXW, and KYF designed the research studies. SYF, JL, YXL, and WGS conducted experiments. SYF, YXL, and WGS acquired data. SYF, JL, YXL, RRW, AUY, YXW, and KYF analyzed data. YXW and KYF provided reagents. SYF wrote the manuscript. JL, YXL, RRW, AUY, YXW, and KYF edited the manuscript. All authors gave final approval and agree to be accountable for all aspects of the work.

## Supplementary Material

Supplemental data

## Version 1 09/29/2022

In-Press Preview

## Version 2 11/08/2022

Electronic publication
