# Supplementary material for: Increased joint loading induces subchondral bone loss of the temporomandibular joint via the RANTES-CCRs-Akt2 axis
Source: JCI Insight. 2022 Nov 8;7(21):e158874. doi: 10.1172/jci.insight.158874 (PMC9675482; doi:10.1172/jci.insight.158874)
Supplement: Supplemental data [file jciinsight-7-158874-s264.pdf]

SUPPLEMENTARY FILES

Supplementary Table 1

Supplementary Table 2

Supplementary Table 3

Supplementary Table 4

Supplementary Figure 1

Supplementary Figure 2

Supplementary Figure 3

Supplementary Figure 4

Supplementary Figure 5

Supplementary Figure 6

Supplementary Figure 7

Supplementary Figure 8

Supplementary Figure 9

Supplementary Table 1

Modified OARSI scoring system

Grade	Criteria
0	The surface of cartilage is intact and continuous. Matrix is evenly distributed without expansion and deformation. Chondrocytes arrange in appropriate orientation in layers with clear borders without signs of proliferation or death assessed by appearing of cell-free area.
1	The surface of cartilage is intact and continuous but uneven. The border of each cartilage layer is still clear, continuous and intact. Chondrocytes are nonuniform. The cells of the proliferative layer proliferate actively. The border between the cartilage and subchondral bone is clear. Add 0.5 if many cells appear in the superficial layer or the cell-free areas (larger than the area occupied by three simulated hypertrophic cells) are found in the proliferative layer.
2	The cartilage becomes thinner. The surface of cartilage is intact and continuous but the border of each cartilage layer is not clear. Add 0.5 if there is cell-free area or clustered proliferation.
3	The cartilage becomes more thinner, and the hypertrophic layer becomes discontinuous and/or thin to only one or two cells layer, or even interrupted locally. The cationic stain depletion of the matrix is over 1/3 or the cells in the proliferative layer proliferate actively. More cells appear in the chondroblastic layer. Add 0.5 if the cell-free area.
4	The cartilage becomes even thinner. The border of each cartilage layer is not clear. Locally some chondrocytes clustered protruding into the subchondral bone. The proliferative layer becomes thin to one or two cells layer, adjacent to the hypertrophic layer. The cationic stain depletion of the matrix is less than 2/3. The calcification zone becomes obvious. Add 0.5 if the cell-free area is found in the chondroblastic layer or in the proliferative layer, or cells proliferate actively in these two layers.
5	The local cartilage becomes thin to 1 or 2 layers of prehypertrophic cells. However, the superficial layer is intact. The number of cells reduced significantly. The cationic stain depletion of the matrix is over 2/3 depth of the whole cartilage.
6	The cartilage becomes thin locally to only superficial layer and proliferative layers with reduced cell numbers, leading the fibrous and proliferative zones of the cartilage adjacent directly to the subchondral bone and cartilage deformation.

Supplementary Table 2

Synovitis scoring system

(1) Synovial lining hyperplasia	
Grade	Criteria
0	Staining of 1-3 layers
1	Staining of 4-6 layers
2	Staining of 7 or more layers
(2) Inflammatory infiltrate	
Grade	Criteria
0	No inflammatory infiltrate
1	Few mostly perivascular situated lymphocytes or plasma cells
2	Numerous lymphocytes or plasma cells, sometimes forming follicle-like aggregates
3	Dense band-like inflammatory infiltrate or numerous large follicle-like aggregates
(3) Fibrin deposits	
Grade	Criteria
0	Not deposit
1	Involving less than one-third of the synovial membrane length
2	Involving one-third to two-thirds of the synovial membrane length
3	Involving more than two-thirds of the synovial membrane length
(4) Vascularity	
Grade	Criteria
0	A limited number (less than 5) of blood vessel profiles/mm^2
1	Focal occurrence of 5-10 small blood vessel profiles/mm^2
2	Focal occurrence of a large number (more than 10) of small blood vessel profiles/mm^2

Supplementary Table 3

Primer pairs used in RT-qPCR analysis

Gene	Forward Primer (5'-3')	Reverse Primer (5'-3')
MMP9	CAAAGACCTGAAAACCTCCAAC	GACTGCTTCTCTCCCATCATC
TRAP	CAAGAACTTGCGACCATTGTTA	ATCCATAGTGAAACCGCAAGTA
CTSK	GCTTGGCATCTTTCCAGTTTTA	CAACACTGCATGGTTCACATTA
GAPDH	GGTTGTCTCCTGCGACTTCA	TGGTCCAGGGTTTCTTACTCC

Supplementary Table 4

Demographic information of the human subjects

	DDw/oR without OA	DDw/oR with early-stage OA	DDw/oR with late-stage OA	*P*-value
Gender				
Female	13	15	12	0.530*
Male	1	0	1	
Age (years)				
Mean ± SD	26.0 ± 8.6[a,b]	23.0 ± 8.0[a]	32.6 ± 8.1[b]	0.013#
Range	13-40	13-39	22-44	
Race (Asian/others)	14/0	15/0	13/0	-

** Results of Chi-square test and post-hoc Z-tests with Bonferroni correction (where applicable).*

# Results of one-way ANOVA with Bonferroni's multiple comparison test. Different letter indicates statistical significance, and same later indicates non-statistical significance between groups.

DDw/oR; disc displacement without reduction. OA: osteoarthritis.

Supplementary Figures

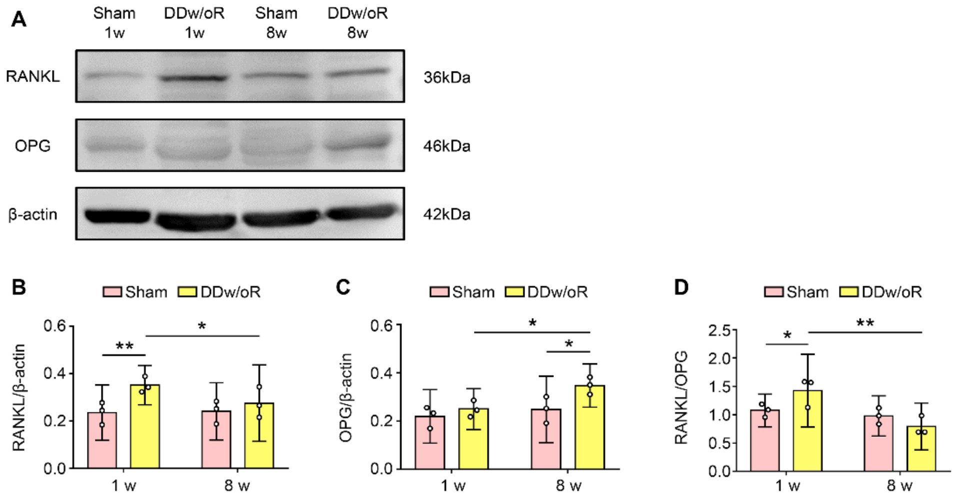

Supplementary Figure 1. Expression levels of RANKL and OPG in subchondral bone of rat TMJOA model induced by DDw/oR. Related to Figure 3. Male SD rats underwent sham or right-sided unilateral DDw/oR surgery. Rats were euthanized at 1-week and 8-week after surgery. **(A)** Representative Western blot bands showing the expression of RANKL, OPG, and β-actin in condylar subchondral bone. **(B)** Quantitative analysis of the relative intensity of RANKL. **(C)** Quantitative analysis of the relative intensity of OPG. β-actin was used as an internal control. **(D)** Quantitative analysis of the ratio of RANKL to OPG. Data information: Data were presented as mean ± 95% confidence interval and showed one representative image of n = 3 independent samples per group. Statistical analyses were determined by two-way ANOVA with Bonferroni's multiple comparison test. * *P* < 0.05. ** *P* < 0.01. Abbreviations: TMJ, temporomandibular joint; DDw/oR, disc displacement without reduction; OA, osteoarthritis.

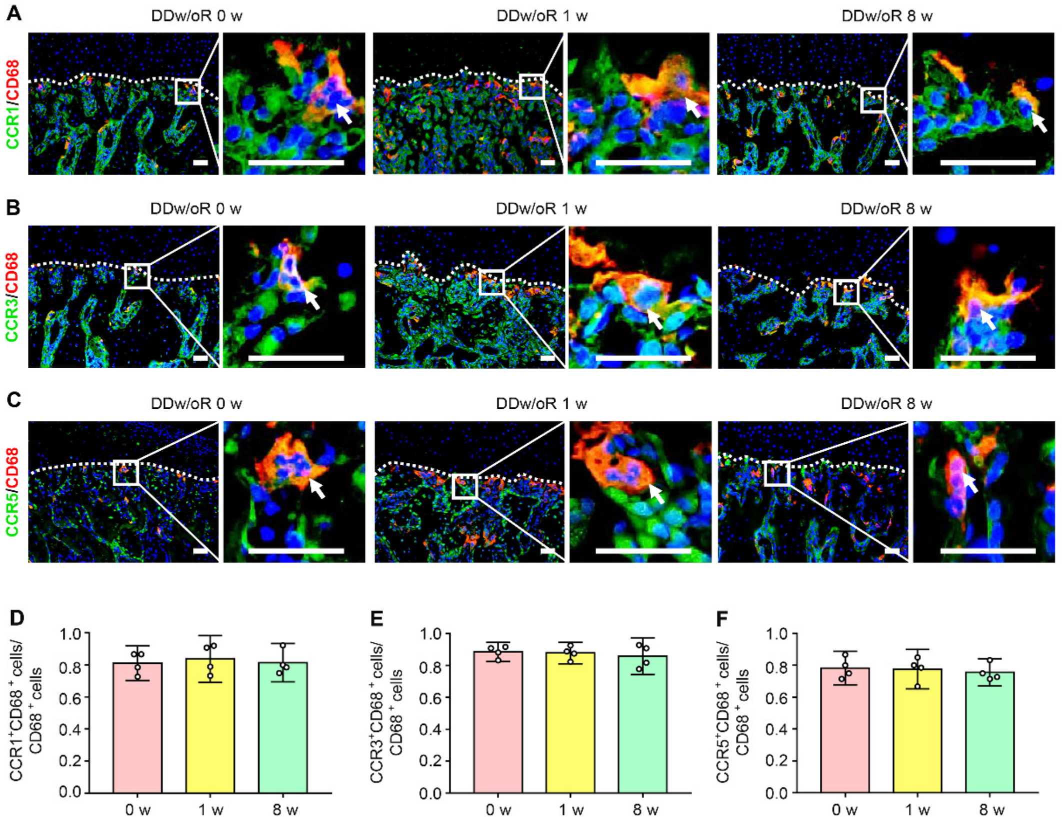

Supplementary Figure 2. CCRs are expressed in macrophages of subchondral bone. Related to Figure 4. Male SD rats underwent right-sided unilateral DDw/oR surgery. Rats were euthanized at baseline (0-week), early (1-week) and late (8-week) time points after surgery. **(A-C)** Representative images of (A) CCR1, (B) CCR3, or (C) CCR5 and CD68 immunofluorescence co-staining in TMJ sagittal sections. CCR1, CCR3, and CCR5 positive cells were presented in green and CD68 positive cells were presented in red. The white dotted line represents the demarcation between articular cartilage and subchondral bone. The white arrows indicate CCR1 CCR3, or CCR5 and CD68 double-positive cells in subchondral bone. Scale bar: 50 μm. **(D-F)** Quantitative analysis of the ratio of (D) CCR1, (E) CCR3, or (F) CCR5 and CD68 double-positive cells to CD68 positive cells in subchondral bone. Data information: Data were presented as mean ± 95% confidence interval and showed one representative image of n = 4 independent samples per group. Statistical analyses were determined by one-way ANOVA with Bonferroni's multiple comparison test. Abbreviations: DDw/oR, disc displacement without reduction.

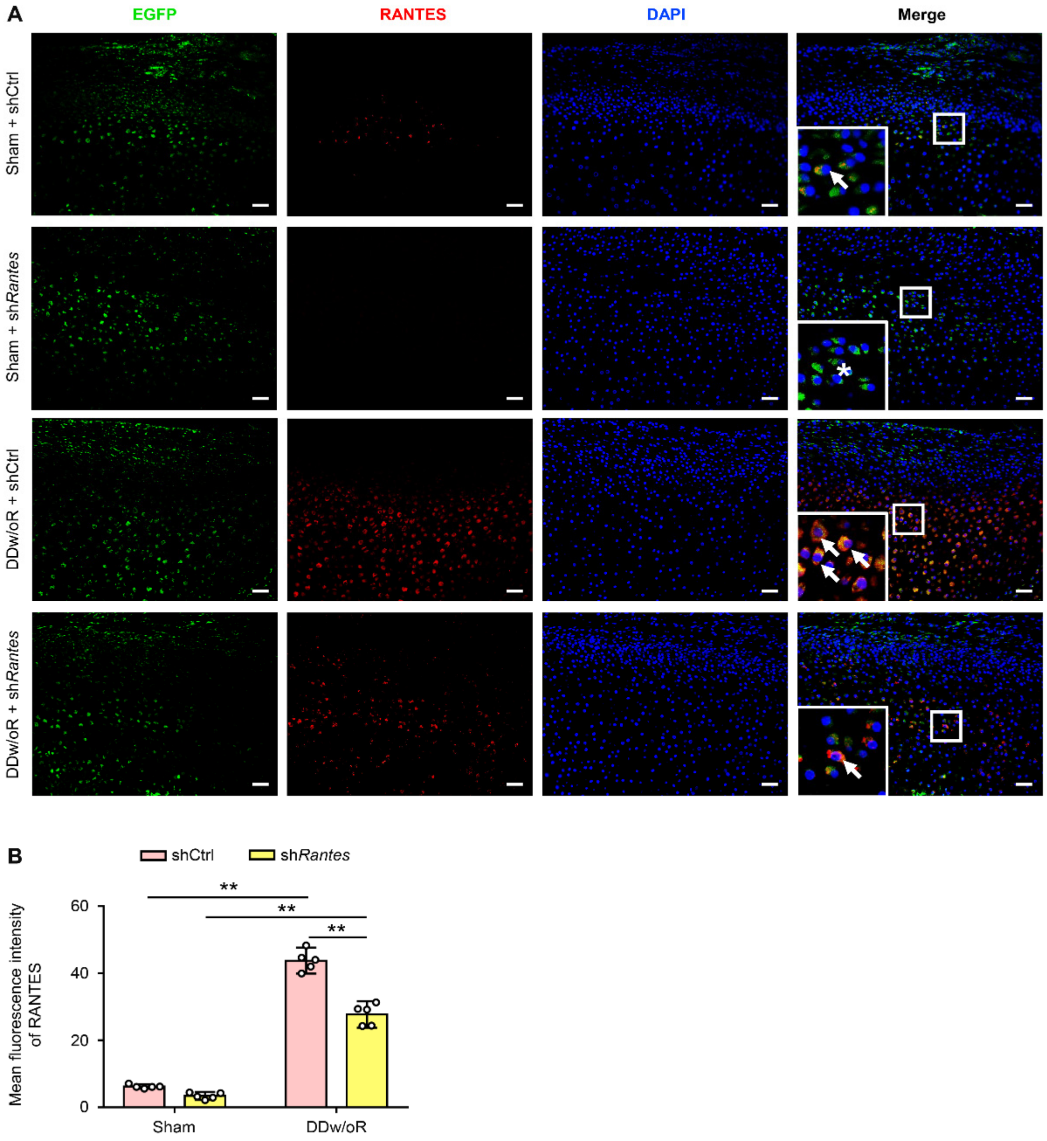

Supplementary Figure 3. The efficiency of sh*Rantes* examined via immunofluorescence staining. Related to Figure 6. Male SD rats underwent sham or right-sided unilateral DDw/oR surgery. Rats were euthanized at 1-week after surgery. **(A)** Representative images of immunofluorescence staining of EGFP and RANTES in articular cartilage in shCtrl- or sh*Rantes*-treated rats. The white arrowheads indicate RANTES$^+$ cells, and asterisks indicate negative signals. Scale bar: 50 μm. **(B)** Quantitative analysis of the mean fluorescence intensity of RANTES$^+$ cells of articular cartilage. Data information: Data were presented as mean ± 95% confidence interval and showed one representative image of n = 5 independent samples per group. Statistical analysis was determined by two-way ANOVA with Bonferroni's multiple comparison test. ** P < 0.01. Abbreviations: TMJ, temporomandibular joint; DDw/oR, disc displacement without reduction.

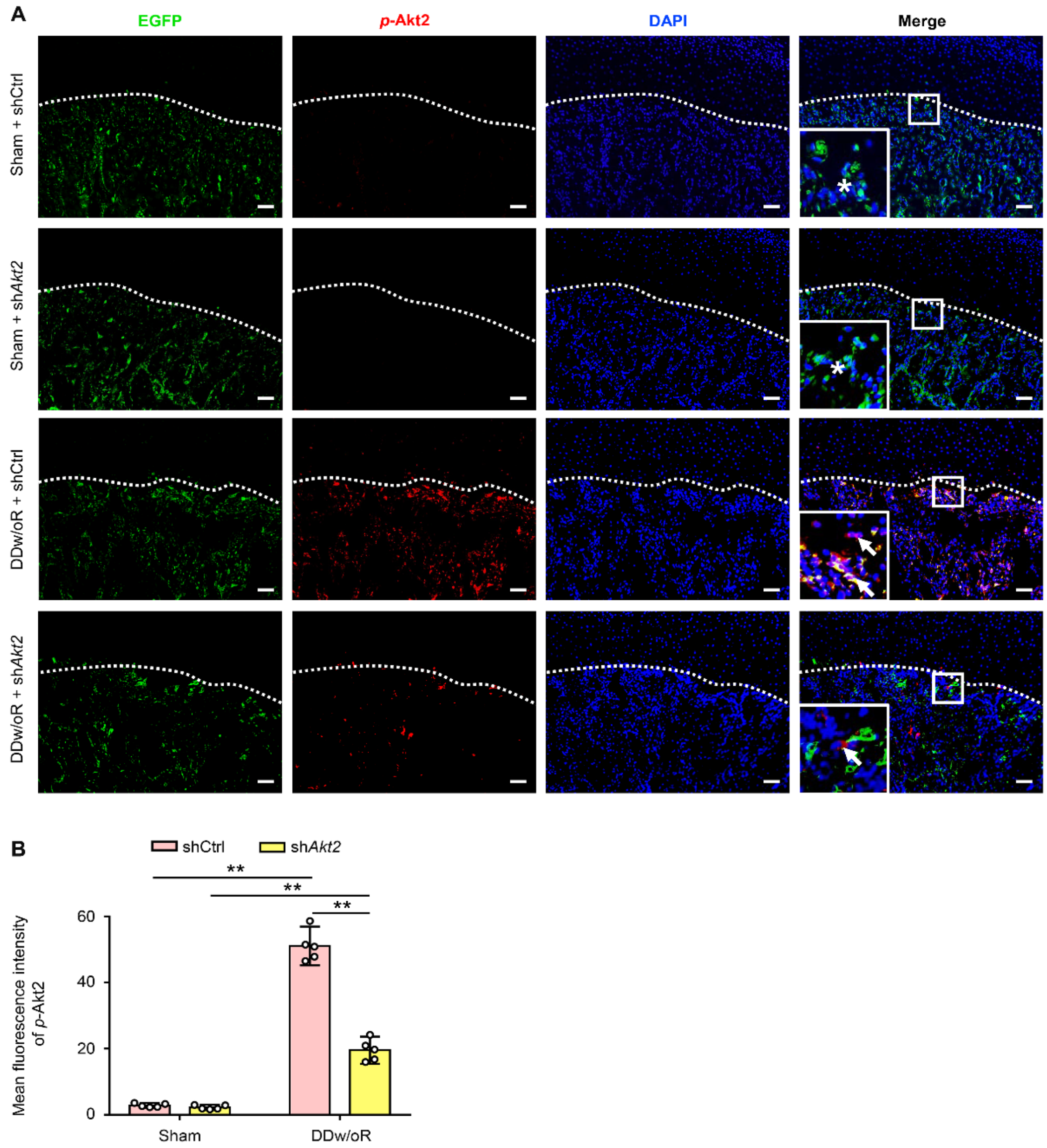

Supplementary Figure 4. The efficiency of sh*Akt2* examined via immunofluorescence staining. Related to Figure 7. Male SD rats underwent sham or right-sided unilateral DDw/oR surgery. Rats were euthanized at 1-week after surgery. **(A)** Representative images of immunofluorescence staining of EGFP and *p*-Akt2 in condyles in shCtrl- or sh*Akt2*-treated rats. The white dotted line shows the demarcation between the articular cartilage and underlying subchondral bone. The white arrowheads indicate *p*-Akt2^{+} cells, and asterisks indicate negative signals. Scale bar: 50 μm. **(B)** Quantitative analysis of the mean fluorescence intensity of *p*-Akt2^{+} cells of condyles. Data information: Data were presented as mean ± 95% confidence interval and showed one representative image of n = 5 independent samples per group. Statistical analysis was determined by two-way ANOVA with Bonferroni's multiple comparison test. ** $P < 0.01$. Abbreviations: TMJ, temporomandibular joint; DDw/oR, disc displacement without reduction.

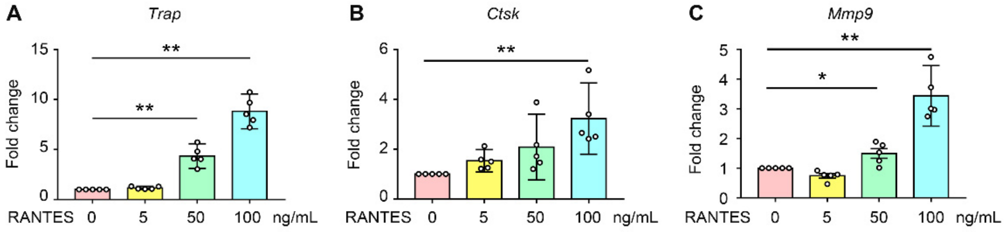

Supplementary Figure 5. RANTES facilitates osteoclast formation in vitro. Related to Figure 10. RAW264.7 cells were treated with different osteoclast-inducing conditions in vitro. **(A-C)** Relative expression of (A) *Trap*, (B) *Ctsk*, and (C) *Mmp9* mRNA levels. Data information: Data were presented as mean ± 95% confidence interval. n = 5 independent experiments with biological replicates. Statistical analyses were determined by one-way ANOVA with Bonferroni's multiple comparison test. * $P < 0.05$. ** $P < 0.01$.

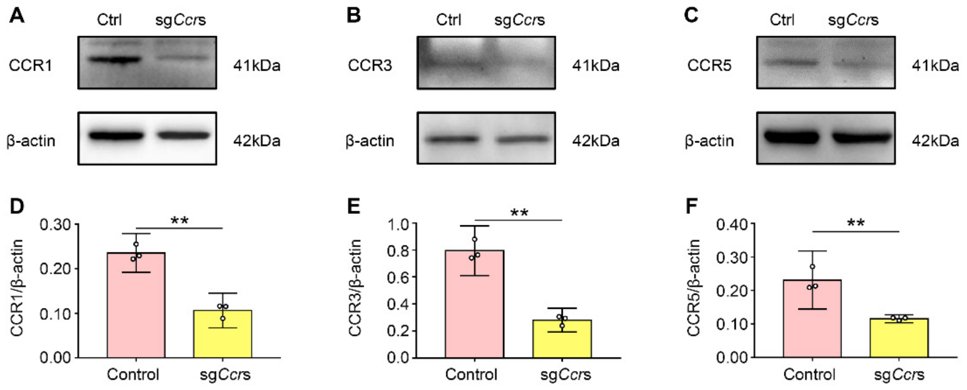

Supplementary Figure 6. The transfection efficiency of sg*Ccr*s checked via Western blot. Related to Figure 10. RAW264.7 cells were treated with different osteoclast-inducing conditions in vitro. **(A-C)** Representative Western blot bands showing the expression of (A) CCR1, (B) CCR3 and (C) CCR5 in RAW 264.7 cells in the control and sg*Ccr*s-treated groups. **(D-F)** Quantitative analysis of the relative intensity of (D) CCR1, (E) CCR3 and (F) CCR5. All levels were normalized to β-actin. Data information: Data were presented as mean ± 95% confidence interval and showed one representative image of n = 3 independent experiments with biological replicates. Statistical analyses were determined by Student's *t*-test. ** $P < 0.01$.

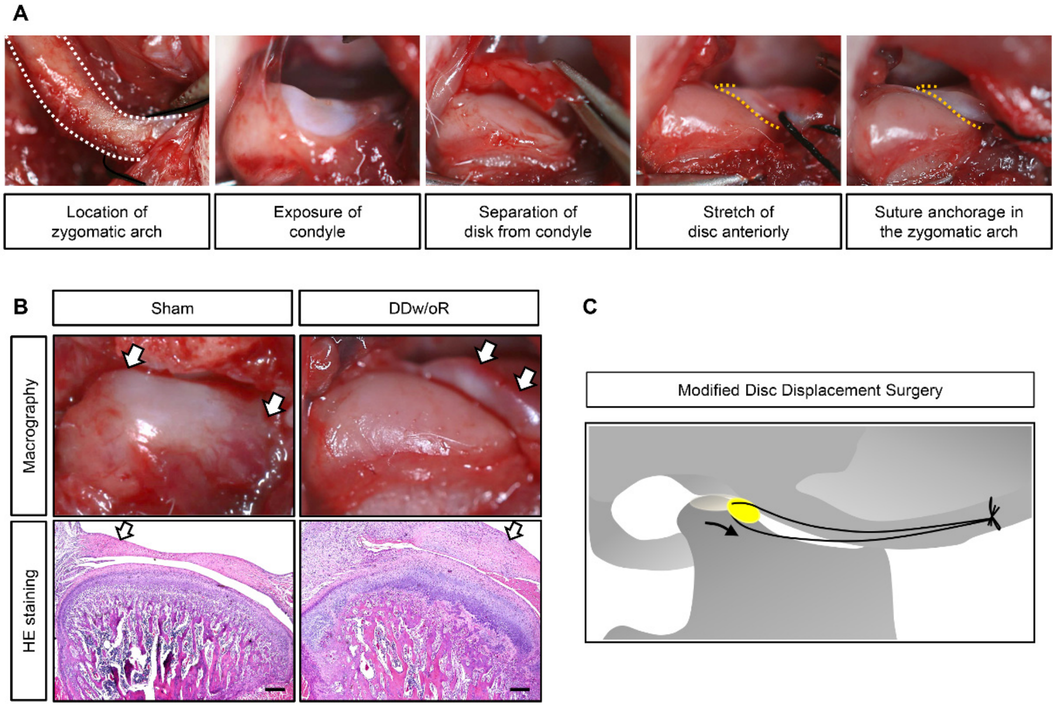

Supplementary Figure 7. Surgical procedure for anterior disc displacement in a rat TMJOA model. Male SD rats underwent sham or right-sided unilateral DDw/oR surgery. **(A)** Steps of the anterior disc displacement procedure. The white dotted line shows the contour of the zygomatic arch. The yellow dotted line shows the posterior margin of the articular disc. **(B)** Macrography and HE staining of the disc-condyle position. The white arrows show the position of TMJ disc. Scale bar: 200 μm. **(C)** Schematic illustration of the modified rat DDw/oR procedure, showing the suture passing through the posterior band of the disc, stretching the disc anteriorly away from the articular surface and anchored in the zygomatic arch in a sagittal view. Abbreviations: TMJ, temporomandibular joint; DDw/oR, disc displacement without reduction; OA, osteoarthritis.

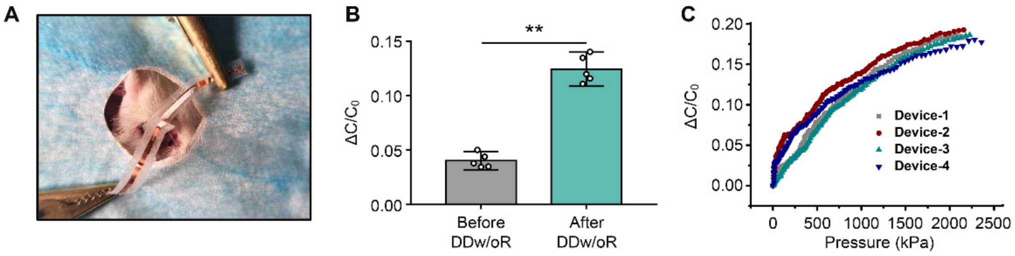

Supplementary Figure 8. Increased pressure on the anterior surface of condyles is yielded by TMJ disc displacement. Related to Figure 8. Male SD rats underwent right-sided unilateral DDw/oR surgery. **(A)** The capacitance-based force sensing system for pressure measurement on the anterior surface of condyles in situ. **(B)** Capacitance variations on the anterior surface of condyles at the maximum passive mouth opening position before and after DDw/oR surgery. **(C)** The pressure-capacitance sensing curves of the capacitance-based force sensing system. Data information: Data were presented as mean ± 95% confidence interval. n = 5 independent samples per group. Statistical analysis was determined by Student's *t*-test. ** *P* < 0.01. Abbreviations: TMJ, temporomandibular joint; DDw/oR, disc displacement without reduction.

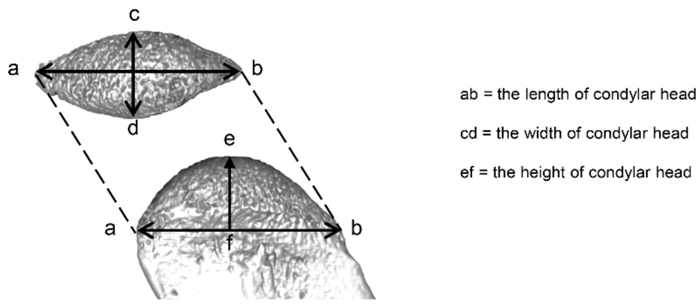

Supplementary Figure 9. Three-dimensional linear measurement of TMJ condylar heads.

Full unedited gel for Figure 5A

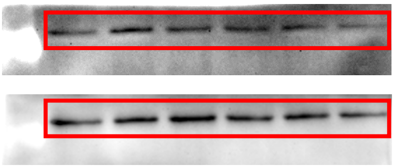

Full unedited gel for Figure 5C

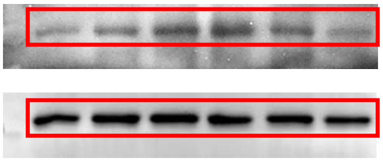

Full unedited gel for Figure 5E

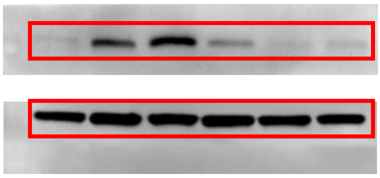

Full unedited gel for Figure 10A

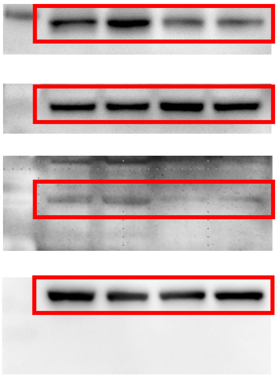

Full unedited gel for Figure 10D

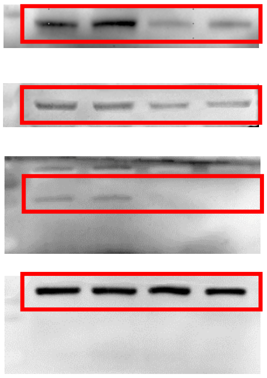

Full unedited gel for Supplementary Figure 1A

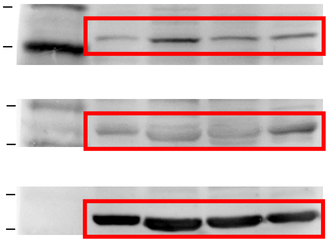

Full unedited gel for Supplementary Figure 6A

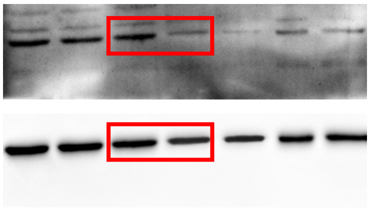

Full unedited gel for Supplementary Figure 6B

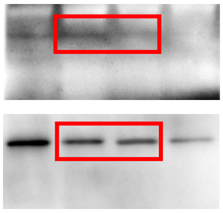

Full unedited gel for Supplementary Figure 6C

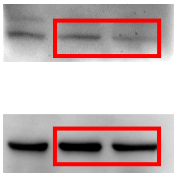

For Western blot, proteins were transferred electrophoretically to a full polyvinylidene fluoride membrane. And then, we cut the membrane into two parts according to different targeted protein sizes. This is our original Western blot images.
